# Moving Beyond Cyanoarene Thermally Activated Delayed Fluorescence Compounds as Photocatalysts: An Assessment of the Performance of a Pyrimidyl Sulfone Photocatalyst in Comparison to 4CzIPN

**DOI:** 10.1021/acs.joc.2c01137

**Published:** 2022-07-12

**Authors:** Megan Amy Bryden, Francis Millward, Tomas Matulaitis, Dongyang Chen, Marco Villa, Andrea Fermi, Sultan Cetin, Paola Ceroni, Eli Zysman-Colman

**Affiliations:** † Organic Semiconductor Centre, EaStCHEM School of Chemistry, University of St Andrews, St Andrews, Fife KY16 9ST, United Kingdom; ‡ Department of Chemistry Ciamician, University of Bologna, Via Selmi 2, 40126 Bologna, Italy; § Center for Chemical Catalysis−C3, University of Bologna, via Selmi 2, 40126 Bologna, Italy

## Abstract

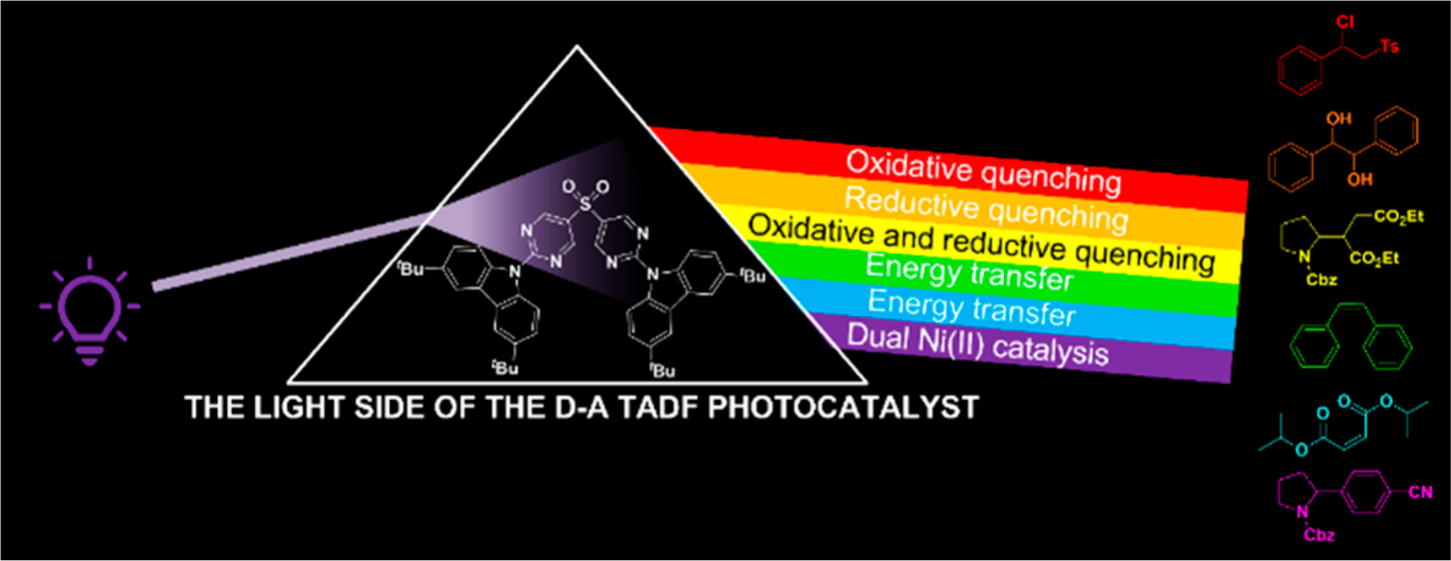

Carbazolyl dicyanobenzene (CDCB) derivates exhibiting thermally activated delayed fluorescence (TADF) have shown themselves to be excellent photocatalysts over recent years, particularly **4CzIPN**, although investigation into organic TADF compounds as photocatalysts outside of the CDCB group has been limited. Herein, we report an alternative donor–acceptor TADF structure, 9,9′-(sulfonylbis(pyrimidine-5,2-diyl))bis(3,6-di-*tert*-butyl-9H-carbazole), **pDTCz-DPmS**, for use as a photocatalyst (PC). A comparison of the electrochemical and photophysical properties of **pDTCz-DPmS** with **4CzIPN** in a range of solvents identifies the former as a better ground state reducing agent and photoreductant, while both exhibit similar oxidation capabilities in the ground and excited state. The increased conjugation of **pDTCz-DPmS** relative to **4CzIPN** presents a more intense CT band in the UV–vis absorption spectrum, aiding in the light absorption of this molecule. Prompt and delayed emission lifetimes are observed for **pDTCz-DPmS**, confirming the TADF nature, both of which are sufficiently long-lived to participate in productive photochemistry. These combined properties make **pDTCz-DPmS** useful in photocatalysis reactions, covering a range of photoredox oxidative and reductive quenching reactions, as well as those involving a dual Ni(II) cocatalyst, alongside energy transfer processes. The higher triplet energy and increased photostability of **pDTCz-DPmS** compared with **4CzIPN** were found to be advantages of this organic PC.

## Introduction

Over the last two decades, photoredox catalysis has become a widespread and useful tool in organic synthesis.^1−3^ This is in part due to the use of much milder reaction conditions in photocatalysis in comparison to the typically higher temperatures and stoichiometric use of reductants, some of which are toxic, often required in traditional thermal synthesis. Additionally, photocatalysis provides alternative and new chemoselectivity, allowing a route to synthons not easily accessible using other synthetic methodologies.^4,5^ As such, photocatalysis has received a resurgence of interest, with just under 17 000 papers published on this topic between 2020 and 2021 combined.^6^

Typically, transition metal complexes based on ruthenium(II) or iridium(III) metals have been the most popular photocatalysts used in homogeneous photocatalysis on account of their well understood and desirable photophysical properties, including their visible-light absorption, long-lived excited states, and versatile redox potentials that can be easily tuned through variation of the ligand field around the metal.^7^ However, issues related to the toxicity, natural abundance of these metals, and associated costs have motivated the search for alternative photocatalysts, with both earth-abundant metal complexes^8^ and organic compounds^3^ identified as potentially viable options for their replacement.

**Figure 1:**
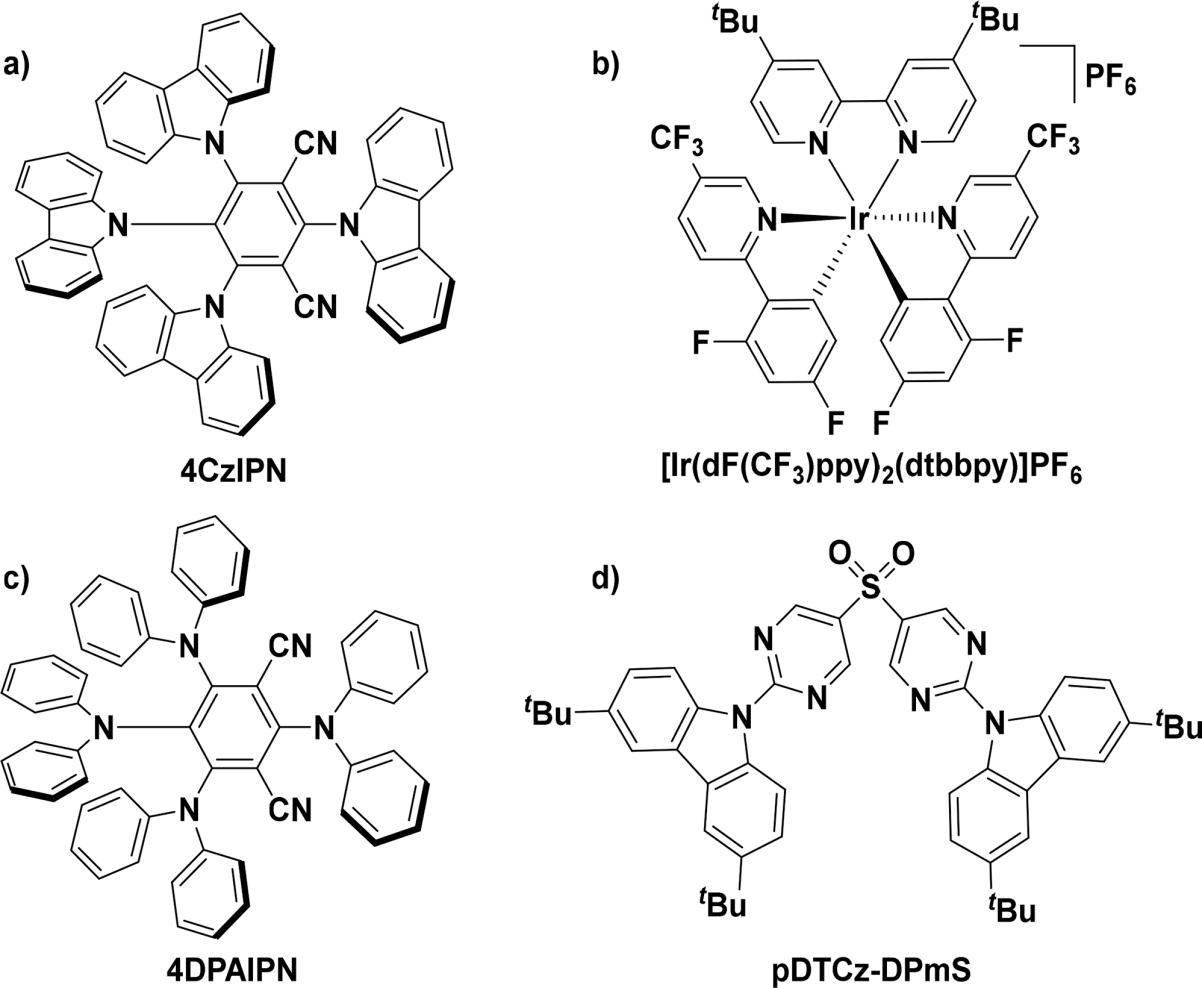
Structures of (a) **4CzIPN**, (b) [Ir(dF(CF_3_)ppy)_2_(dtbbpy)]PF_6_, (c) **4DPAIPN**, and (d) **pDTCz-DPmS**.

Particularly, since 2016 the organic compound 2,4,5,6-tetra(9*H*-carbazol-9-yl)isophthalonitrile, **4CzIPN**, (Figure 1a) has become a popular choice as a photocatalyst.^9−11^ First developed as an emitter for organic light-emitting diodes (OLEDs),^12^ this compound possesses remarkably similar photophysical properties to the commonly used [Ir(dF(CF_3_)ppy)_2_(dtbbpy)]PF_6_ [(dF(CF_3_)ppy) = 2-(2,4-difluorophenyl)-5-(trifluoromethyl)pyridinato and dtbbpy = 4,4′-di-*tert*-butyl-2,2′-bipyridine] (Figure 1b).^13,14^**4CzIPN** absorbs into the visible region of the electromagnetic spectrum (λ_abs_ = 435 nm in MeCN),^11^ has a long-lived excited state lifetime as it shows thermally activated delayed fluorescence (TADF), (τ_p_ = 18.7 ns and τ_d_ = 1390 ns in MeCN,^15^ where τ_p_ and τ_d_ refer to the prompt and delayed fluorescence lifetimes, respectively), and possesses a suitable range of redox potentials (Table 1). **4CzIPN** is composed of an isophthalonitrile acceptor core, decorated with four carbazole electron donor moieties. The steric interactions between adjacent carbazole groups create large torsions between the donor groups to the isophthalonitrile unit. The resultant highly twisted structure ensures that the highest occupied molecular orbital (HOMO) is localized on the donor groups while the lowest unoccupied molecular orbital (LUMO) is located on the acceptor phthalonitrile moiety, leading to a small exchange integral and a correspondingly small singlet–triplet excited state energy gap, Δ*E*_ST_. The suitably small Δ*E*_ST_ is required for the observed TADF associated with this compound.^16^

**Table 1:** Selected Optoelectronic Properties of **4CzIPN** and **pDTCz-DPmS**^a^

compound	λ_abs_ (nm)	λ_PL_ (nm)	*E*_0,0_ (eV)	Δ*E*_ST_ (eV)	τ_p_ (ns)	τ_d_ (μs)	*E*_ox_ (V)	*E*_red_ (V)	*E*_ox_^*^ (V)	*E*_red_^*^ (V)
4CzIPN	448	544	2.60	0.12^b^	24.6^b^	2.04^b^	1.51	–1.21	–1.09	1.39
pDTCz-DPmS	363	524	3.01	0.27	3.0	3.4	1.57	–1.67	–1.44	1.34

a Values are reported in dichloromethane (DCM). *E*_0,0_ determined from the intersection point between the normalized absorption and emission spectra. Δ*E*_ST_ was calculated as the difference of the first singlet (*E*_S1_) and first triplet (*E*_T1_) excited state energies (Δ*E*_ST_ = *E*_S1_ – *E*_T1_), estimated from the onsets of the prompt fluorescence and phosphorescence spectra at 77 K, respectively. τ_p_ and τ_d_ refer to the prompt and delayed fluorescence lifetimes, respectively. Redox potentials are reported vs SCE and are obtained from the maxima of the oxidation and reduction waves in the DPV. *E*_ox_^*^ = *E*_ox_ – *E*_0,0_ and *E*_red_^*^= *E*_red_ + *E*_0,0_.

b Values taken from ref (15) in DCM.

A particular benefit of donor–acceptor organic TADF compounds such as **4CzIPN** lies in the facility to modulate their optoelectronic properties through modification of the structures of the donor or acceptor units, which, due to their relatively small electronic coupling, impact mostly independently the HOMO and LUMO levels, respectively.^17^ This facile tuning of the photophysical and electrochemical properties is an attractive and beneficial quality, one that historically has been challenging to achieve with organic photocatalysts.^18,19^ Moreover, organic TADF photocatalysts present an opportunity to access both the singlet and triplet excited states for photochemistry, whether this be through an electron or energy transfer mechanism.^9^ In terms of electron transfer, accessing the singlet excited state is advantageous in that it is a more potent oxidant and reductant than the corresponding more stabilized triplet state, the latter of which is only available to heavy transition metal complexes. For energy transfer photocatalysis, organic TADF compounds have the ability to participate in both Förster and Dexter energy transfer, while again, heavy metal complexes are typically limited to only Dexter energy transfer.

To date, there are now over 200 reports that demonstrate the use of **4CzIPN** and related donor–acceptor compounds, such as **4DPAIPN** (Figure 1c), as effective photocatalysts.^9,20^ Despite the thousands of examples of organic TADF compounds used as emitters in OLEDs,^17^ very few of these compounds have been investigated as potential photocatalysts.^9^ Recently, imidazoacridine-based TADF compounds were shown to be effective energy transfer photocatalysts in [2 + 2] cycloadditions.^21^ The work of Kwon et al.^22^ is notable as they computationally explored a wide variety of donor–acceptor architectures as potential photocatalysts in polymerization reactions. Their study revealed that a selection of donor–acceptor compounds incorporating a sulfone acceptor unit showed useful photocatalytic activity in these reactions.

Inspired by this report, we identified a donor–acceptor sulfone-containing compound, 9,9′-(sulfonylbis(pyrimidine-5,2-diyl))bis(3,6-di-*tert*-butyl-9H-carbazole), **pDTCz-DPmS**, (Figure 1d), that we had previously developed as a TADF emitter in OLEDs,^23^ for use as a potential photocatalyst. This compound possesses a wider ground state redox window, a longer delayed lifetime, and is significantly more (photo)reducing than **4CzIPN** (Table 1). An initial version of this work was deposited in ChemRxiv on May 6th, 2022.^24^

## Results and Discussion

### Electrochemical and Photophysical Characterization

The relevant electrochemical and photophysical data of **pDTCz-DPmS** were first ascertained in a range of different polarity solvents to reflect the medium used in the photocatalytic testing (vide infra) and these results were cross-compared with density functional theory (DFT) calculations (vide infra). The solvents tetrahydrofuran (THF), dichloromethane (DCM), *N*,*N*-dimethylformamide (DMF), and acetonitrile (MeCN) were chosen for investigation due to their frequent use as the medium in photocatalytic reactions.

The DFT calculations conducted on **pDTCz-DPmS** in the aforementioned solvents indicated that little to no change would be observed when changing solvent polarity, the S_1_ predicted energy remained at 3.47 eV in all solvents, while the T_1_ energy increased from 2.97 eV in THF and DCM to 2.98 eV in the more polar DMF and MeCN (Table S8). Despite the theoretical Δ*E*_ST_ value being too large to be considered TADF (0.50 eV in THF and DCM or 0.49 eV in DMF and MeCN), these results were consistent with our previous gas-phase DFT calculations on this molecule,^23^ and it was later confirmed in our subsequent measurements that **pDTCz-DPmS** does indeed display TADF character when dissolved in these solvents, with much smaller experimental Δ*E*_ST_.

Cyclic voltammetry (CV) and differential pulse voltammetry (DPV) measurements for **pDTCz-DPmS** and **4CzIPN** permitted the determination of the ground and excited state redox potentials, thermodynamic parameters that are of greatest relevance to photoredox catalysis to assess the feasibility of the single electron transfer (SET) events. Measurements were obtained in THF, DCM, DMF, and MeCN; due to poor solubility in MeCN, no measurements were possible in this solvent for **pDTCz-DPmS**. The solvent windows for THF and DMF are limited in the oxidation range, hence the oxidation potentials could not be determined in these solvents.

The CVs of **pDTCz-DPmS** show both chemically irreversible oxidation and reduction waves, while **4CzIPN** exhibits an irreversible oxidation wave and reversible reduction wave in most solvents (Figure S5 of the Supporting Information, SI). The redox potentials for both compounds are provided in Table S1. While the ground state oxidation potentials in DCM of **4CzIPN** and **pDTCz-DPmS** are similar at 1.51 V and 1.57 V, respectively, the latter compound exhibits a significantly more negative ground state reduction potential (−1.21 V and −1.67 V, respectively, in DCM), implying that **pDTCz-DPmS** will be a more effective ground state reducing agent. Small variations of up to 50 mV in *E*_red_ are obtained for both compounds as a function of solvent polarity. Generally, with increasing solvent polarity, a more negative *E*_red_ value is observed. Due to the small electrochemical windows of THF and DMF, the impact of solvent polarity on *E*_ox_ could not be determined.

**Figure 2:**
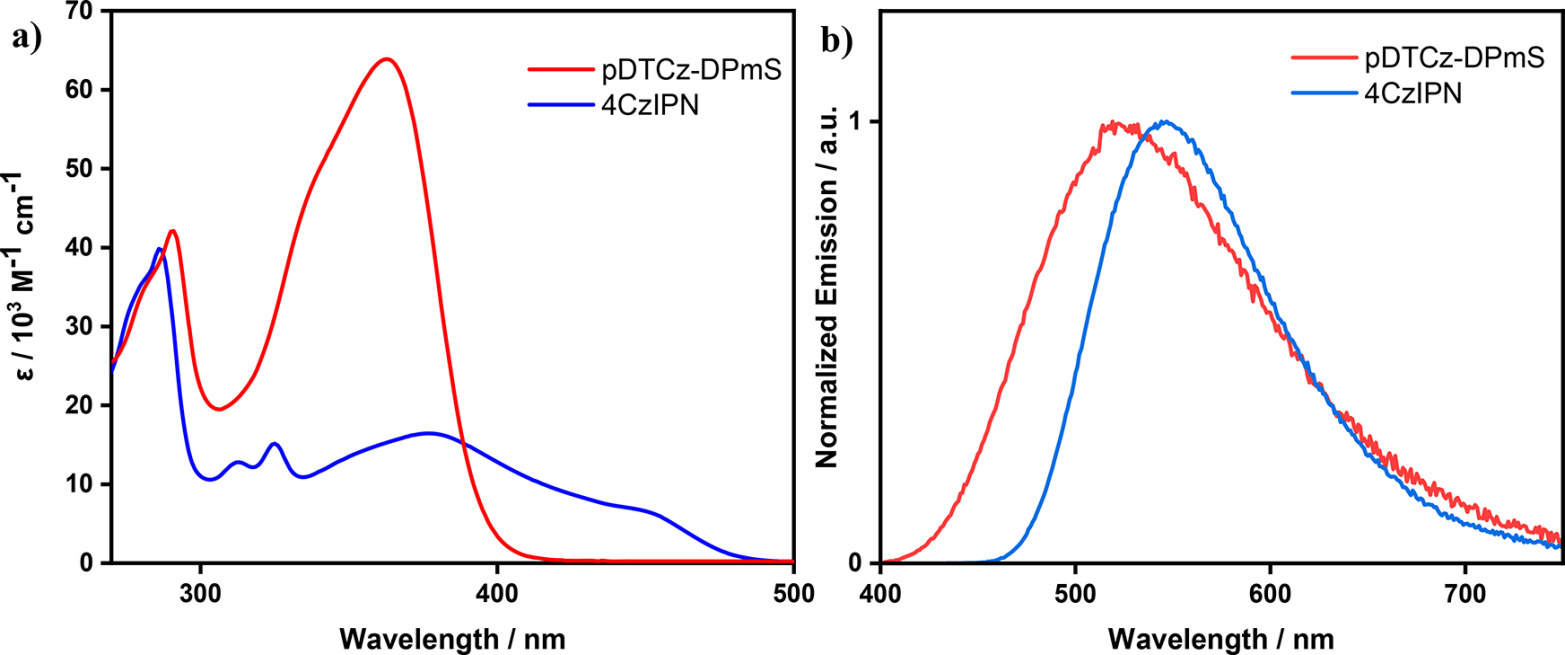
(a) UV–vis absorption spectra and (b) PL spectra for **4CzIPN** and **pDTCz-DPmS** in DCM. λ_exc_ = 420 nm for **4CzIPN** and 360 nm for **pDTCz-DPmS**. Measurements performed at room temperature under air.

The UV–vis absorption spectra of **4CzIPN** and **pDTCz-DPmS** are similar, with both compounds possessing low energy charge transfer (CT) bands (λ_abs_ = 448 and 363 nm, respectively, in DCM). Due to the greater conjugation between donor and acceptor in **pDTCz-DPmS**, which is a result of its more planar conformation, these CT bands are much more intense (Figure 2a). The onset of absorption is significantly more red-shifted in **4CzIPN** in comparison to **pDTCz-DPmS**. There is a limited degree of negative solvatochromic effect observed for both compounds (Figure S6), reflecting a decrease in the transition dipole moment of the compounds in the excited state in these solvents.

The normalized steady-state photoluminescence (PL) spectra of **4CzIPN** and **pDTCz-DPmS** in DCM are presented in Figure 2b. The Gaussian band shape, alongside the observed positive solvatochromism (Figure S6), provide evidence of the charge transfer (CT) character of the emissive excited state. The optical gap, *E*_0,0_, identified from the intersection point between the normalized absorption and emission spectra (Table S2) are 2.60 and 3.01 eV for **4CzIPN** and **pDTCz-DPmS**, respectively, in DCM. **pDTCz-DPmS** displays a much larger *E*_0,0_ than **4CzIPN**, irrespective of solvent. This is due to both the emission of **pDTCz-DPmS** being slightly blue-shifted relative to **4CzIPN** (λ_PL_ = 524 and 544 nm in DCM, respectively) as well as the red-shifted CT absorption band present in **4CzIPN** compared to that in **pDTCz-DPmS**. As a result, **pDTCz-DPmS** has an optical gap ∼0.5 eV larger than that of **4CzIPN**; thus, **pDTCz-DPmS** has a wider excited state redox window (Table S1). The excited state reduction potentials are relatively similar, regardless of solvent choice (*E*_red_^*^ = 1.34 and 1.39 V for **pDTCz-DPmS** and **4CzIPN** in DCM, respectively), while **pDTCz-DPmS** is a considerably stronger photoreductant than **4CzIPN** (*E*_ox_^*^ = −1.44 V and −1.09 V, respectively, in DCM).

The photoluminescence quantum yields of **pDTCz-DPmS**, measured in degassed conditions, are strongly dependent on the solvent polarity with a value of 42% in toluene and 2% in DMF solution (Table 2). In an attempt to better ascertain trends associated with the solvent polarity, toluene was also employed as a solvent for photophysical measurements since it is nonpolar. The photoluminescence quantum yields measured under air-equilibrated solution correspond roughly to the prompt emission and show a similar trend. The ratio of the prompt and delayed emission quantum yield changes from 2:1 in toluene and DMF to almost 5:1 in THF and DCM, without a clear trend evident with respect to the solvent polarity.

**Table 2:** Selected Photophysical Properties for **pDTCz-DPmS** in Toluene, THF, DCM, and DMF^a^

solvent	toluene	THF	DCM	DMF
Φ_PL_ (%)	42 (28)	17 (14)	7.0 (5.8)	2.0 (1.4)
Φ_PROMPT_/Φ_TADF_	2.0	4.7	4.8	2.3
*k*_ISC_ (s^–1^)^b^	6.36 × 10^7^	1.78 × 10^8^	1.17 × 10^8^	3.12 × 10^7^
*k*_RISC_ (s^–1^)^b^	1.39 × 10^5^	2.65 × 10^5^	4.53 × 10^5^	5.19 × 10^5^
τ_p_ (ns) [weighting (%)]	7.3 (54)	3 (59)	3 (68)	4 (89)
τ_d_ (μs) [weighting (%)]	13.4 (46)	8.1 (41)	3.4 (32)	2.2 (11)
*E*_S1_ (eV)	3.20	3.09	3.20	3.13
*E*_T1_ (eV)	2.95	2.93	2.93	2.97
Δ*E*_ST_ (eV)	0.25	0.16	0.27	0.16

a Photoluminescence quantum yields, Φ_PL_, were determined under deaerated conditions while the values in parentheses refer to the air-equilibrated solutions. Prompt and delayed lifetimes (τ_p_ and τ_d_, respectively) were recorded at room temperature under vacuum using λ_exc_ = 378 nm. The first excited singlet (*E*_S1_) and triplet energies (*E*_T1_) were determined by the onset of the room temperature photoluminescence and 77 K phosphorescence spectra, respectively.

b Rates are determined using the method and assumptions outlined in reference (27).

The time-resolved PL decays of **pDTCz-DPmS** were measured for 10^–5^ M solutions under vacuum at room temperature (Figure S7). In toluene, THF, DCM, and DMF, the emission decays with biexponential kinetics (Table 2). The prompt emission occurs with a 3 to 7 ns lifetime (τ_p_) in all four solvents, while the microsecond-range delayed emission lifetime, τ_d_, becomes shorter with increasing solvent polarity. Regardless of solvent, the excited states of both compounds are sufficiently long-lived to participate in photocatalysis. The faster TADF decay of **4CzIPN** can be linked to the larger reverse intersystem crossing (RISC) rate and is indicative of a smaller Δ*E*_ST_ (0.12 and 0.27 eV for **4CzIPN** and **pDTCz-DPmS**, respectively, in DCM).

For **4CzIPN**, Adachi et al. found that with increasing solvent polarity, *k*_ISC_ between S_1_ to T_1_ decreases (from 5.1 × 10^7^ s^–1^ in toluene to 2.2 × 10^6^ s^–1^ in MeCN).^15^ This was hypothesized to be a result of the interaction between the singlet excited state of **4CzIPN** and solvent molecules, which suppressed *k*_ISC_ as has been seen in carbene and fluorenone systems.^25,26^ Indeed, Wang et al. suggested that in the carbene system, the solvated carbene must first be desolvated before ISC can occur, thus causing the *k*_ISC_ to decrease. This trend in *k*_ISC_ was proposed to be the reason behind the decrease in the photoluminescence quantum yield (Φ_PL_) in more polar solvents (94% and 18% for toluene and MeCN, respectively). By contrast, the value of *k*_RISC_ was found to increase with solvent polarity (from 2.7 × 10^6^ s^–1^ in toluene to 1.4 × 10^7^ s^–1^ in MeCN). These findings are similarly reflected in the trends observed for **pDTCz-DPmS** (Table 2).

### Photocatalysis Investigations

**Figure 3:**
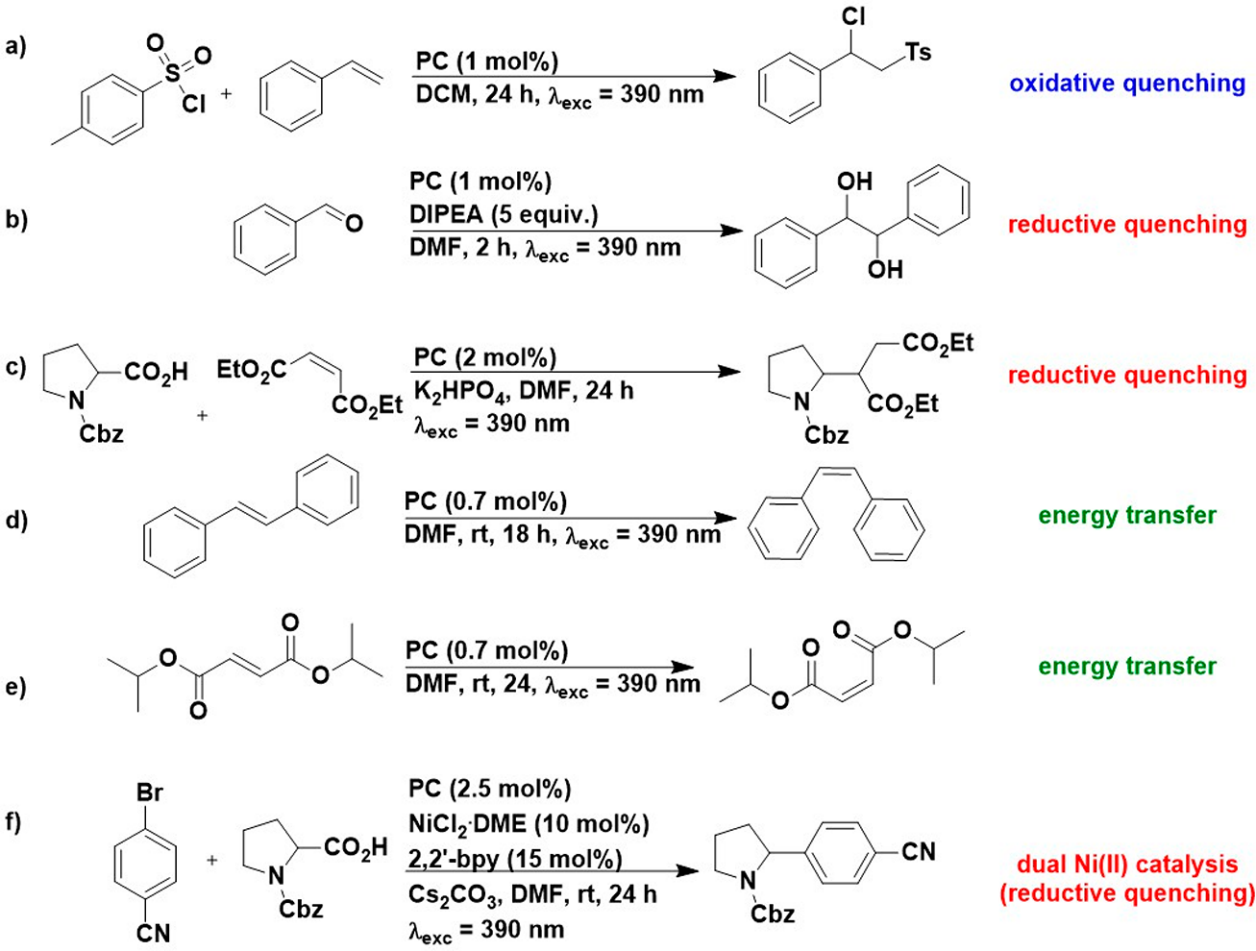
Photocatalysis reactions investigated: (a) oxidative quenching, (b and c) reductive quenching, (d and e) energy transfer, and (f) dual metallaphotocatalysis with a Ni(II) cocatalyst.

The potential of **pDTCz-DPmS** to act as a photocatalyst was subsequently evaluated in a range of prototypical photochemical reactions that cover the suite of commonly encountered mechanisms: reductive quenching, oxidative quenching, energy transfer, and dual metallaphotocatalysis with a Ni(II) cocatalyst (Figure 3). The performance of **pDTCz-DPmS** was cross-compared with that of **4CzIPN** as well as the reference photocatalysts previously reported for these reactions.

### Oxidative Quenching

We first assessed the potential of **pDTCz-DPmS** as a photocatalyst in an atom transfer radical addition (ATRA) reaction with styrene and tosyl chloride (TsCl).^28,29^ Reiser et al. had employed transition metal PCs such as [Cu(dap)_2_]Cl or [Ru(bpy)_3_](PF_6_)_2_, affording under the optimized conditions 96% or 80% of the coupled product, respectively, for the substrates shown in Figure 3a.^30^ The proposed mechanism involves an oxidative quench of the excited PC by TsCl, generating a tosyl radical and a halide anion. The tosyl radical is then proposed to add to the olefin, with the resultant radical being oxidized by the oxidized photocatalyst, closing the photocatalytic cycle. The PC must be sufficiently photoreducing to reduce TsCl (E_red_ = −0.94 V vs SCE)^30^ while being capable in its oxidized form to oxidize the carbon-centered radical intermediate. For the copper PCs, the proposed mechanism also involved coordination of the substrates to the metal center, hence for simplicity, [Ru(bpy)_3_](PF_6_)_2_ was used as the reference PC for comparison as [Cu(dap)_2_]Cl may be implicated in both inner sphere and outer sphere electron transfer chemistry.

The literature yield of [Ru(bpy)_3_](PF_6_)_2_ obtained by Reiser et al. using 455 nm CREE XP LEDs as the light source and MeCN as the solvent for 24 h could be replicated using our photocatalytic setup (80% vs 81%, respectively) when matching the conditions as closely as possible, with the use of a 456 nm Kessil lamp being the only significant change. However, these conditions needed to be altered to 390 nm irradiation in DCM due to the absorption profile and solubility of **pDTCz-DPmS**. Under these conditions, the yield obtained from [Ru(bpy)_3_](PF_6_)_2_ dropped to 64%. On the basis of the redox potentials (Table 3), we envisaged that both **4CzIPN** and **pDTCz-DPmS** could promote this transformation, particularly the latter. Unfortunately, while both could photocatalyze the reaction, they do so in poor yields of 10% and 16%, respectively, with a considerable amount of unreacted styrene detected in the reaction mixture by ^1^H NMR spectroscopy. It is unclear at this point why the organic TADF photocatalysts perform so much more poorly than [Ru(bpy)_3_](PF_6_)_2_.

**Table 3:** Average ^1^H NMR Yields Obtained in the ATRA Reaction and Relevant Redox Potentials of the Photocatalysts^a^

photocatalyst	*E*_ox_ (V)	*E*_ox_^*^ (V)	^1^H NMR yield (%)
[Ru(bpy)_3_](PF_6_)_2_	1.42	–0.86	64 ± 3(80)^b^
4CzIPN	1.51	–1.09	10 ± 1
pDTCz-DPmS	1.57	–1.44	16 ± 2

a Redox potentials reported vs SCE and in DCM unless otherwise noted. Values in parentheses indicate the ^1^H NMR yield obtained in the literature. Reaction conditions followed are those shown in Figure 3 (refer to SI for further details).

b Value taken from ref (30) using 455 nm irradiation and MeCN as the solvent.

### Reductive Quenching

We next assessed **pDTCz-DPmS** as a photocatalyst in two reductive quenching reactions: the pinacol coupling of benzaldehyde (Figure 3b); as well as the decarboxylative addition of *N*-Cbz-Pro to diethyl maleate (Figure 3c). In the former, the proposed mechanism involves reductive quenching of the PC by di*iso*propylethylamine (DIPEA), followed by reduction of benzaldehyde by the reduced PC, facilitated by the presence of the Lewis acidic radical cation of DIPEA.^31^ The iridium PC, [Ir(ppy)_2_(dtbbpy)]PF_6_, provided a reported yield of 44% under the conditions of Rueping et al. involving 11 W 450 nm LEDs with MeCN as the solvent for 15 h, which is matched using our setup (Table 4) utilizing 390 nm Kessil lamps, DMF as the solvent and a reaction time of only 2 h.

**Table 4:** Average ^1^H NMR Yields Obtained for the Reductive Quenching Reactions and Relevant Redox Potentials of the Photocatalysts^a^

photocatalyst	*E*_red_ (V)	*E*_red_^*^ (V)	reaction	^1^H NMR yield (%)
[Ir(ppy)_2_(dtbbpy)]PF_6_	–1.42	1.08	Pinacol coupling	43 ± 3
				74 ± 3^b^
				(44)^c^
4CzIPN	–1.24	1.40	Pinacol coupling	68 ± 0
				76 ± 3^b^
pDTCz-DPmS	–1.62	1.48	Pinacol coupling	32 ± 1
				80 ± 3^b^
[Ir(dF(CF_3_)ppy)_2_(dtbbpy)]PF_6_	–1.27	1.46	decarboxylative addition	99 ± 0
				(93)^d^
4CzIPN	–1.24	1.40	decarboxylative addition	99 ± 0
				(80)^e^
pDTCz-DPmS	–1.62	1.48	decarboxylative addition	64 ± 3

a Redox potentials reported vs SCE and in DMF unless otherwise noted. Values in parentheses indicate the ^1^H NMR yield obtained in literature unless otherwise noted. Yields for the pinacol reaction refer to combined yield of the meso:dl isomers. Reaction conditions followed are those shown in Figure 3 unless otherwise noted (refer to SI for further details).

b Reaction run for 24 h.

c Value taken from ref (31) using MeCN as the solvent, 2 equiv. NBu_3_ (in replacement of DIPEA) and 450 nm irradiation for 15 h.

d Value taken from ref (34) using 26 W CFL and is an isolated yield using dimethyl maleate as the substrate.

e Value taken from ref (18) using 455 nm LEDs in MeCN and is an isolated yield.

Pleasingly, **pDTCz-DPmS** photocatalyzed this reaction but again, despite ostensibly having a larger thermodynamic driving force, the NMR yield falls short of that obtained for **4CzIPN** (Table 4) when conducting the reaction for a period of 2 h. This may be linked to the molar absorptivity, ε, of the two photocatalysts at 390 nm, of which the ε for **4CzIPN** is higher (14.9 × 10^3^ M^–1^ cm^–1^ vs 12.8 × 10^3^ M^–1^ cm^–1^, for **4CzIPN** and **pDTCz-DPmS**, respectively, see Figure 2a). Since ε is linked to the rate of reaction,^32^ the low yield may be explained by slower reaction kinetics; note that the reaction time is only 2 h, following the conditions of Wenger et al.^33^ Increasing the reaction time to 24 h resulted in a much larger reaction yield for **pDTCz-DPmS** whereas there was only a minor difference in yield when **4CzIPN** was used as the PC (Table 4), resulting in comparable yields for the two PCs. Under our conditions, the reaction was also found to proceed without the need for a photocatalyst, this is termed the background reaction. Although the background reaction does also increase with the longer time period (Table S4), this cannot fully account for the increase in yield for **pDTCz-DPmS**. Notably, PCs such as [Ru(bpy)_3_](PF_6_)_2_ cannot turn over this transformation as documented in the literature^31,33^ and reproduced with our setup.

**Scheme 1:**
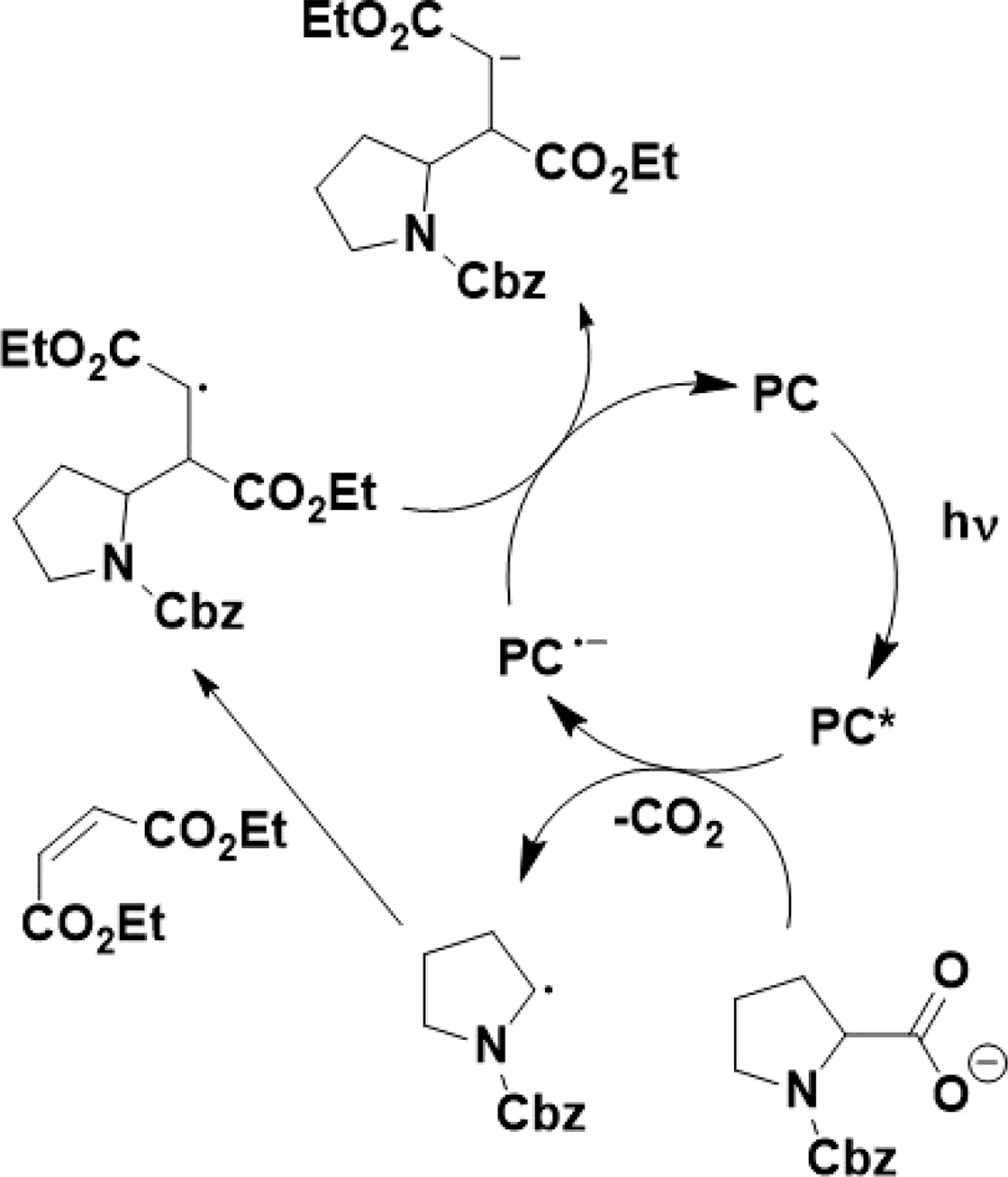
Literature Reported Proposed Mechanism for the Decarboxylative Addition of *N*-Cbz-Pro to Diethyl Maleate^,^^35^ Reproduced from *J. Am. Chem. Soc.*, **2014**, *136* (14), 5257–5260. Copyright 2014 American Chemical Society.

The decarboxylative addition of *N*-Cbz-Pro to diethyl maleate (Figure 3c) was selected as a model reaction to investigate oxidative photocatalysis through a reductive quenching cycle; both [Ir(dF(CF_3_)ppy)_2_(dtbbpy)]PF_6_ and **4CzIPN** have been shown to be highly effective and both of them were described to be reductively quenched by the deprotonated form of *N*-Cbz-Pro.^18,34^ According to these literature reports, the PC must be capable of first oxidizing the *N*-Cbz-Pro carboxylate (*E*_ox_ = 0.68 V vs SCE in DMF for *tert-*butylammonium *N*-Cbz-Pro salt, Figure S17) as well as being suitably reducing in the ground state to reduce the in situ generated α-acyl radical, as depicted in Scheme 1. The literature yield of 93% obtained using [Ir(dF(CF_3_)ppy)_2_(dtbbpy)]PF_6_ could be replicated in our setup (Table 4), while with **4CzIPN** as the PC, the yield obtained was higher than that reported by Zeitler et al. (99% vs 80%, respectively).

**pDTCz-DPmS** could also photocatalyze this reaction but at a lower yield of 64% (Table 4). We investigated the mechanism of the photoreaction to provide insight into the origin of the differences in yields compared to the previously reported photocatalysts. We did not observe any quenching of the prompt fluorescence upon addition of the reagents. To our surprise, we observed a strong quenching of the delayed fluorescence of **pDTCz-DPmS** in degassed DMF solution upon addition of diethyl maleate (*k*_q_ = 7.0 × 10^8^ M^–1^ s^–1^, Figure S11). Upon addition of *N*-Cbz-Pro, quenching was observed only in the presence of K_2_HPO_4_ and only after a few hours stirring (due to the limited solubility of the base in DMF). The efficiency of quenching of **pDTCz-DPmS** was evaluated at the same concentrations used in the reaction conditions for diethyl maleate and *N*-Cbz-Pro (in the presence of K_2_HPO_4_): 85% of the excited states are deactivated by diethyl maleate quenching, 8% by *N*-Cbz-Pro quenching and 7% decays by intramolecular processes (see SI for more details). In comparison, for **4CzIPN** quenching is observed only for *N*-Cbz-Pro in the presence of K_2_HPO_4_ and only after a few hours stirring (Figure S12). No quenching is observed after addition of diethyl maleate.

On the basis of the reduction potential of diethyl maleate (*E*_red_ = −1.43 V vs SCE in DMF, Figure S17), oxidative quenching of **pDTCz-DPmS** (*E*_ox_^*^= −1.44 V vs SCE in DCM) by diethyl maleate is thermodynamically feasible, while this is not the case for **4CzIPN** (*E*_ox_^*^= −1.09 V vs SCE in DCM). To ensure the quenching of **pDTCz-DPmS** by diethyl maleate was occurring through SET, and not by energy transfer via a *Z* → *E* isomerization process, the irradiation of diethyl maleate in the presence of **pDTCz-DPmS** was conducted. As expected from the reported triplet energies of the maleate and fumarate isomers (*E*_T_ = 3.08 and 2.87 eV, respectively),^36^**pDTCz-DPmS** (*E*_T_ = 2.97 in DMF) could not isomerize diethyl maleate (see SI for more details), therefore we can confidently conclude that this quenching process proceeds via SET.

**Scheme 2:**
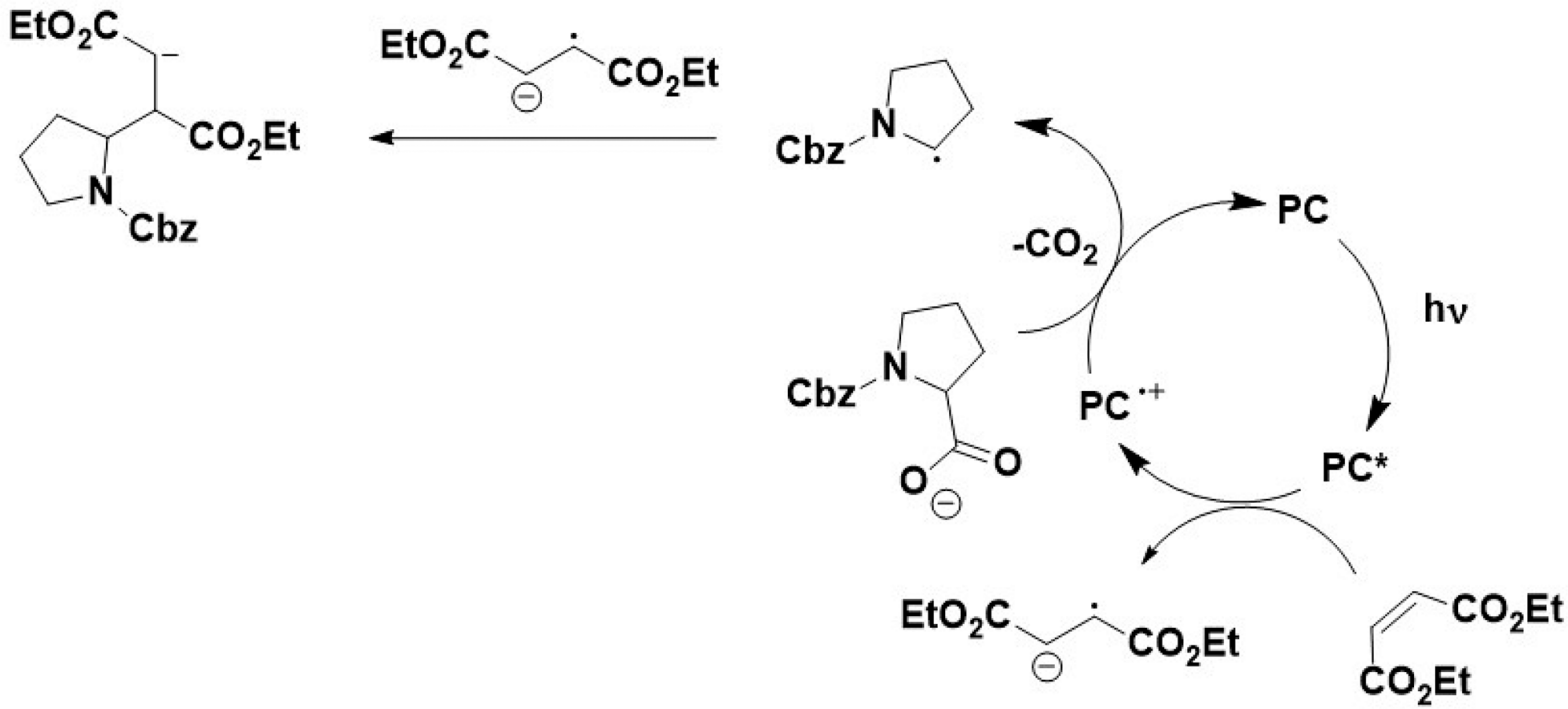
Proposed Reaction Mechanism for the Decarboxylative Addition of Diethyl Maleate to *N*-Cbz-Pro When Using **pDTCz-DPmS** as a Photocatalyst Based on Our Experimental Observations

Combining these experimental observations prompted us to propose an alternative (Scheme 2) and competitive mechanism to that outlined in Scheme 1.

We also contend that photosubstitution of **4CzIPN** under the reaction conditions may additionally play a role in the yields obtained. Indeed, König et al. have recently reported that dicyanobenzene-based PCs undergo photosubstitution of one of the cyano groups when irradiated in the presence of carboxylic acids and base.^37^ The resultant photosubstituted product is significantly more photoreducing, in part based on the larger *E*_0,0_ evidenced by the blue shift of the absorption spectrum. On the basis of this report, we assessed the photostability of **4CzIPN** and **pDTCz-DPmS** in the presence of *N*-Cbz-Pro and base (Figures S13 and S14), which revealed the photosubstitution of **4CzIPN** while **pDTCz-DPmS** is photostable. The photosubstitution experiment was repeated under the exact reaction conditions, namely in the presence of a radical trap in the form of diethyl maleate. Again, the photosubstitution of **4CzIPN** was observed, while **pDTCz-DPmS** remained photostable (Figures S15 and S16).

### Energy Transfer

Having demonstrated the potential of **pDTCz-DPmS** as a photoredox catalyst in reactions that proceed by both oxidative and reductive quenching mechanisms, we next explored this compound in the context of an energy transfer photocatalytic reaction. We first investigated the *E/Z* isomerization of stilbene (Figure 3d), following the conditions employed by Zhang et al.^38^ Reaction yields are shown in Table 5. The photocatalytic isomerization of alkenes is a well-documented process^39−41^ that proceeds via a Dexter energy transfer mechanism. For Dexter energy transfer to be operational, there must be orbital overlap between the donor and acceptor, which can be achieved through bimolecular collisions in an intermolecular reaction. Additionally, spectral overlap between the emission of the energy donor (the photocatalyst) and absorption of the energy acceptor (the *E*-alkene) is required. Triplet energy levels of the PC and the substrate are typically used to crudely assess whether the energy transfer is thermodynamically feasible. To prevent photocatalyzed isomerization of the *Z*-alkene back to the *E*-alkene, the triplet state of the PC must be of intermediate energy to those of the configurational isomers. For stilbene, the *E*_T_ are 2.2 and 2.5 eV, respectively, for the *E* and *Z* isomers.^42^ In the photocatalyzed isomerization, the *E*-isomer can be selectively photoexcited to its triplet state, forming a triplet diradical intermediate that is then free to rotate to form the thermodynamically less stable *Z*-isomer.

**Table 5:** Average ^1^H NMR Yields Obtained in the E/Z Isomerization Reaction and Triplet Energy Level of the Photocatalysts^a^

photocatalyst	*E*_T_ (eV)	alkene	^1^H NMR yield (%)
[Ru(bpy)_3_](PF_6_)_2_	2.13^d^	*E*-stilbene	81 ± 1
			(87)^c^
[Ir(dF(CF_3_)ppy)_2_(dtbbpy)]PF_6_	2.65^b^	di*iso*propyl fumarate	58 ± 1
			(88:12)
4CzIPN	2.59^e^	*E*-stilbene	87 ± 1
			(87:13)^c^
		di*iso*propyl fumarate	6 ± 1
			(0:100)^c^
pDTCz-DPmS	2.97	*E*-stilbene	63 ± 4
		di*iso*propyl fumarate	81 ± 2

a Triplet energy level reported in DMF and obtained at 77 K unless otherwise noted. Values in parentheses indicate the ^1^H NMR yield or the *Z*/*E* ratio obtained in the literature. Reaction conditions followed are those shown in Figure 3 (refer to SI for further details).

b Value taken from ref (44) and was determined in MeCN from the emission maximum.

c Values taken from ref (38) using a 26 W CFL as the irradiation source.

d Value taken from ref (45) and determined in an ethanol:methanol (4:1 v/v) glass at 77 K.

e Value taken from ref (46) and was determined in 2-methyltetrahydrofuran at 77 K.

When using *E*-stilbene as the substrate, **pDTCz-DPmS** successfully forms the isomeric product, although it only does so in a moderate yield of 63% while for **4CzIPN**, the yield of the *Z* isomer is higher at 87%. This is a result of **4CzIPN** possessing a more suitable *E*_T_ to sensitize the *E*-isomer while the *E*_T_ for **pDTCz-DPmS** is considerably higher, and thus there is a lack of chemoselectivity to selectively sensitize only the *E*-isomer. By contrast, when exploring the *E/Z* isomerization of di*iso*propyl fumarate (Figure 3e), a higher triplet energy alkene (*E*_T_ = 2.7 and 3.1 eV, respectively, for the *E* and *Z* isomers),^43^**pDTCz-DPmS** provides a significantly greater yield than **4CzIPN** (81% and 6%, respectively), as well as outperforming the literature photocatalyst [Ir(dF(CF_3_)ppy)_2_(dtbbpy)]PF_6_ (58%). In this example, a higher photocatalyst *E*_T_ level is required to efficiently sensitize the substrate, which **pDTCz-DPmS** can provide, while **4CzIPN** cannot. The *E*/*Z* directionality of the di*iso*propyl fumarate isomerization process is facilitated by the stabilizing n_O_ → π_C=O_* interaction of the *Z*-isomer, which reduces the conjugation of the product chromophore and raises the triplet energy of the maleate isomer.^36^

### Dual Catalysis with a Ni(II) Cocatalyst

Finally, we investigated the potential of **pDTCz-DPmS** in a commonly used Ni-cocatalyzed metallaphotocatalysis reaction involving the cross-coupling of carboxylic acids with aryl halides (Figure 3f).^11^ The proposed mechanism involves the reductive quenching of the excited PC by the carboxylate to yield an alkyl radical following decarboxylation. Closure of the photocatalytic cycle occurs by SET from the reduced PC to the Ni(I) species. Additionally, the PC is proposed to be responsible for the in situ generation of the active Ni(0) species through two SET reductions (*E*_red_(Ni^II^/Ni^0^) = −1.2 V vs SCE in DMF).^47^ As a result, the PC must be moderately photooxidizing as well as sufficiently reducing in the ground state.

**pDTCz-DPmS** afforded the coupled product in a yield of 72%, although this is lower than the quantitative 99% yield obtained with **4CzIPN** (Table 6). Zhang et al. also found that despite having appropriate redox potentials, some donor–acceptor PCs failed to perform as well as **4CzIPN**, which they tentatively attributed to their inferior photochemical stability under the reaction conditions.^11^ We suspect this is again linked to a combination of the photosubstitution of **4CzIPN** (in contrast to the photostability of **pDTCz-DPmS**) as shown in Figures S13–S16, as well as the possibility of an alternative oxidative quenching mechanism being in operation, as previously discussed for reaction **3c**.

**Table 6:** Average ^1^H NMR Yields Obtained in the Dual Catalysis Reaction and Relevant Redox Potentials of the Photocatalysts^a^

photocatalyst	*E*_red_ (V)	*E*_red_^*^ (V)	^1^H NMR yield (%)
4CzIPN	–1.24	1.40	99 ± 1
			(82)^b^
pDTCz-DPmS	–1.62	1.48	72 ± 4

a Redox potentials reported vs SCE in DMF unless otherwise noted. Values in parentheses indicate the isolated yield obtained in the literature. Reaction conditions followed are those shown in Figure 3 (refer to SI for further details).

b Value taken from ref (11) using *N*-Boc-Pro irradiated by a 26 W CFL for 10 h and is an isolated yield.

## Conclusions

We have identified a donor–acceptor TADF compound that can perform competitively in a range of photocatalytic reactions with the commonly used **4CzIPN**, encompassing a variety of different mechanisms. In the aforementioned results, the ability of **pDTCz-DPmS** to act as a PC in comparison to **4CzIPN** is consistently explored. Given the much stronger photoreducing capacity of **pDTCz-DPmS**, relative to **4CzIPN**, we envisage that the former will be a much more potent PC in reactions that proceed via an oxidative quenching mechanism, particularly when an alternative reductive quenching pathway is not possible. Moreover, **pDTCz-DPmS** stands to be an effective candidate for photoinduced energy transfer reactions for substrates with challengingly high triplet energies, due to its higher *E*_T_ of ∼0.4 eV compared to **4CzIPN**. Indeed, **pDTCz-DPmS** has triplet energy more reflective of benzophenone (*E*_T_ = 3.00 eV), a well-known efficient energy transfer photocatalyst.^48^ The much-improved photostability of **pDTCz-DPmS** relative to **4CzIPN** under the reaction conditions investigated presents an additional advantage of this compound. As such, our study shows that TADF donor–acceptor compounds beyond **4CzIPN** and the CDCB family can not only be employed but also can perform even more efficiently than this popular organic photocatalyst.

## Supporting Information Available

Synthetic procedures, NMR spectra, electrochemistry, details of DFT calculations (including coordinates of optimized structures), luminescence studies, and photocatalysis setup (PDF)

## Notes

The research data supporting this publication can be accessed at https://doi.org/10.17630/831e6094-2936-49af-985d-110f16e23f6a.
