# Supplementary material for: Moving Beyond Cyanoarene Thermally Activated Delayed Fluorescence Compounds as Photocatalysts: An Assessment of the Performance of a Pyrimidyl Sulfone Photocatalyst in Comparison to 4CzIPN
Source: J Org Chem. 2022 Jul 12;88(10):6364–73. doi: 10.1021/acs.joc.2c01137 (PMC10204087; doi:10.1021/acs.joc.2c01137)
Supplement: Supplementary file 1 — jo2c01137_si_001.pdf [file jo2c01137_si_001.pdf]

Supporting Information

Moving Beyond Cyanoarene Thermally Activated Delayed Fluorescence Compounds as Photocatalysts: An Assessment of the Performance of a Pyrimidyl Sulfone Photocatalyst in Comparison to 4CzIPN

Megan Amy Bryden,[a] Francis Millward,[a] Tomas Matulaitis,[a] Dongyang Chen[a] Marco Villa,[b] Andrea Fermi,[b,c] Sultan Cetin,[b] Paola Ceroni[b,c] * and Eli Zysman-Colman[a] *

[a] Organic Semiconductor Centre, EaStCHEM School of Chemistry, University of St Andrews, St Andrews, Fife, U.K., KY16 9ST, Fax: +44-1334 463808; Tel: +44-1334 463826;
E-mail: eli.zysman-colman@st-andrews.ac.uk;
[b]Department of Chemistry Ciamician, University of Bologna, Via Selmi 2, 40126 Bologna, Italy; e-mail: paola.ceroni@unibo.it
[c]Center for Chemical Catalysis–C^3, University of Bologna, via Selmi 2, 40126 Bologna, Italy

Table of contents

Experimental Section S2
Electrochemistry S8
Photophysical measurements S10
Mechanistic studies of the decarboxylative addition reaction S13
Photocatalysis S22
NMR S28
DFT calculations S38
References S50

Experimental Section

General Synthetic Procedures. The following starting materials were synthesised according to literature procedures: **4CzIPN**,[1] **2CzPN**,[2] **[Ru(bpy)$_3$](PF$_6$)$_2$**,[3] **[Ir(dF(CF$_3$)ppy)$_2$(dtbbpy)]PF$_6$**,[4] **[Cu(dap)$_2$]Cl**,[5] **[Cu(dmp)(Xantphos)]PF$_6$**,[6] **(bromoethynyl)benzene**,[7] **1,3-dioxoisoindolin-2-yl cyclohexanecarboxylate**,[8] **Hantzsch ester**[9] and ***N*-Cbz-proline**.[10] All other reagents and solvents were obtained from commercial sources and used as received. Air-sensitive reactions were performed under a nitrogen atmosphere using Schlenk techniques, no special precautions were taken to exclude air or moisture during work-up and crystallisation. Anhydrous THF, DCM, toluene and acetonitrile were obtained from a MBraun SPS5 solvent purification system. Flash column chromatography was carried out using silica gel (Silia-P from Silicycle, 60 Å, 40-63 μm). Analytical thin-layer-chromatography (TLC) was performed with silica plates with aluminum backings (250 μm with F-254 indicator). TLC visualization was accomplished by 254/365 nm UV lamp. ^{1}H spectra were recorded on a Bruker Advance spectrometer (400 or 500 MHz for ^{1}H). The following abbreviations have been used for multiplicity assignments: "s" for singlet, "d" for doublet, "t" for triplet, "q" for quartet, "m" for multiplet, and "br" for broad. ^{1}H spectra were referenced residual solvent peaks with respect to TMS (δ = 0 ppm).

Photophysical measurements. Optically dilute solutions of concentrations on the order of 10^{-5} or 10^{-6} M of the photocatalysts were prepared in spectroscopic or HPLC grade solvents for absorption and emission analysis. Absorption spectra were recorded at room temperature on a Shimadzu UV-2600 double beam spectrophotometer and a Varian Cary 50 BIO spectrophotometer with a 1 cm quartz cuvette or a Hellma ultra-micro cuvette with 3 mm optical path length. Molar absorptivity determination was verified by linear regression analysis of values obtained from five independent solutions at varying concentrations with absorbance ranging from 4.12×10^{-6} to 2.06×10^{-5} M. For emission studies, aerated solutions were bubbled by compressed air for 5 minutes and spectra were taken using the cuvette for absorption analysis. Degassed solutions were prepared via four freeze-pump-thaw cycles and spectra were taken using home-made Schlenk quartz cuvette. Steady-state emission, excitation spectra and time-resolved emission spectra were recorded at 298 K using an Edinburgh Instruments F980 or a Perkin Elmer LS55 spectrofluorometer, equipped with a Hamamatsu R928 phototube. Samples were excited at 360 nm or 420 nm for steady-state measurements and at 378 nm or 340 nm for time-resolved measurements.

The singlet-triplet splitting energy ΔE_{ST} was estimated by recording the prompt fluorescence spectra and phosphorescence emission at 77 K. An open Dewar was used for solution samples. The samples were photoexcited using the third harmonic emission (343 nm) from a femtosecond Nd:YAG laser, which originally emits at 1030 nm (Orpheus-N, model: PN13F1). Emission from the samples was focused onto a spectrograph (Chromex imaging, 250is spectrograph) and detected on a sensitive gated iCCD camera (Stanford Computer Optics, 4Picos) having subnanosecond resolution. Phosphorescence spectra were measured 1 ms after the excitation of the Nd:YAG laser with iCCD exposure time of 8.5 ms. Prompt fluorescence spectra were measured 1 ns after the excitation of the femtosecond laser with iCCD exposure time of 100 ns.

Fitting of time-resolved luminescence measurements: Time-resolved PL measurements were fitted to a sum of exponentials decay model, with chi-squared (χ^2) values between 1 and 2, using the EI FLS980 or Edinburgh FLS920 software. Each component of the decay is assigned a weight, (w_i), which is the contribution of the emission from each component to the total emission.

Emission quantum yield measurements: Emission quantum yields were measured following the method of Demas and Crosby[11] using $[Ru(bpy)_3]^{2+}$ as the standard in air-equilibrated aqueous solution $\Phi = 0.0405$.[12]

Electrochemistry measurements. Cyclic Voltammetry (CV) analysis was performed on an Electrochemical Analyzer potentiostat model 620E from CH Instruments at a sweep rate of 100 mV/s. Differential pulse voltammetry (DPV) was conducted with an increment potential of 0.004 V and a pulse amplitude, width, and period of 50 mV, 0.05, and 0.5 s, respectively. Samples were prepared as acetonitrile (MeCN), dichloromethane (DCM), tetrahydrofuran (THF) or N,N-dimethylformamide (DMF) solutions, which were degassed by sparging with solvent-saturated argon gas for 5 minutes prior to measurements. All measurements were performed using 0.1 M solution of tetra-*n*-butylammonium hexafluorophosphate ($[nBu_4N]PF_6$]). An Ag/Ag^+ electrode was used as the reference electrode while a glassy carbon electrode and a platinum wire were used as the working electrode and counter electrode, respectively. The redox potentials are reported relative to a saturated calomel electrode (SCE) with a ferrocenium/ferrocene (Fc/Fc^+) redox couple as the internal standard (0.38 V vs SCE for MeCN,[13] 0.46 V vs SCE for DCM, 0.56 V vs SCE for THF and 0.45 V vs SCE for DMF).[14]

Theoretical Calculations. All ground state optimizations have been carried out using Density Functional Theory (DFT) level with Gaussian 16[15] using the PBE0 functional[16] and the 6-31G(d,p) basis set,[17] except for triplet excited state optimizations, where calculations at the same level of theory were made using unrestricted DFT. All calculations employed a polarizable continuum model (PCM) to simulate the solvent environment for each of the solvents MeCN, DMF, DCM and THF. Excited state calculations were performed using Time-Dependent DFT (TD-DFT) using the same functional and basis set as for ground state geometry optimization. Calculations were automated using an in-house designed software package, *Silico*, which uses a number of 3rd party libraries and programs, including: extraction and processing of results: cclib,[18] generation of 3D images: VMD[19] & Tachyon,[20] generation of graphs: Matplotlib,[21] calculation of CIE colour coordinates: Colour Science,[22] generation of report: Mako[23] & Weasyprint,[24] scientific constants: SciPy,[25] conversion of file formats: Pybe[26] & Openbabel.[27] Structures were visualized with *Gaussview* v5.0. *GaussSum*3.0 was used to and visualize simulated absorption spectra (full-width at half maximum set to 1000 cm^{-1}). *Chemissian* v4.67 was used to model atom group contributions to the frontier molecular orbitals.

Synthesis

pDTCz-DPmS was synthesised according to a modified literature procedure in 4 steps.[28]

Step 1: Synthesis of 9-(5-bromopyrimidin-2-yl)-3,6-di-*tert*-butyl-9H-carbazole (tCz-BrPm) was completed according to literature procedure.[28]

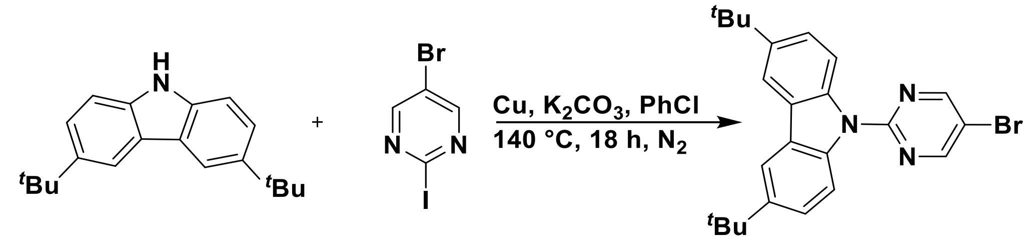

Figure **S1**. Reaction scheme for the synthesis of tCz-BrPm.

To an oven dried flask were added 5-bromo-2-iodopyrimidine (1.57 g, 5.5 mmol, 1 equiv.), di-*tert*-butyl-9*H*carbazole (1.7 g, 6.1 mmol, 1.1 equiv.), copper powder (0.35 g, 5.5 mmol, 1 equiv.) and potassium carbonate (2.28 g, 16.5 mmol, 3 equiv.). The flask was degassed by three cycles of vacuum-nitrogen purging and 12 mL of dry chlorobenzene was injected. The mixture was stirred at 140 °C using an oil bath for 18 h under a nitrogen atmosphere. The reaction mixture was allowed to cool before being then poured into water (30 mL) and extracted with DCM (3 × 20 mL). The combined organic layers were dried over anhydrous $MgSO_4$, filtered and the solvent removed under reduced pressure. The crude product was purified by silica gel column chromatography. DCM:Hexane =1:3 was used as eluent to afford tCz-BrPm as a white solid. **Yield:** 1.70 g, 71%. **R_f:** 0.65 (33% DCM:Hexane). **^{1}H NMR (400 MHz, $CDCl_3$), δ (ppm):** 8.80 (s, 2H), 8.69 (d, 2H), 8.04 (dd, 2H), 7.54 (dd, 2H), 1.46 (s, 18H). The ^{1}H NMR spectrum is consistent with the literature.[28]

Step 2: Synthesis of 9-(5-iodoopyrimidin-2-yl)-3,6-di-*tert*-butyl-9H-carbazole (tCz-IPm)

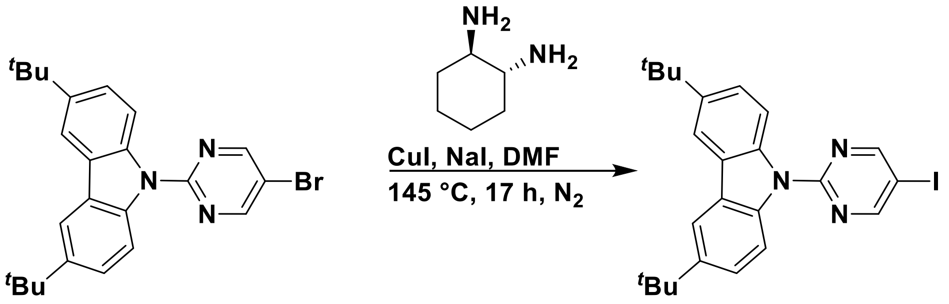

Figure **S2**. Reaction scheme for the synthesis of tCz-Ipm.

To an oven dried flask were added tCz-BrPm (0.67 g, 1.54 mmol, 1 equiv.), NaI (0.915 g, 6.14 mmol, 4 equiv.) and CuI (0.029 g, 0.154 mmol, 0.1 equiv.). The flask was degassed by three cycles of vacuum-nitrogen purging and 24 mL of dry DMF was injected alongside *trans*-1,2-cyclohexanediamine (0.037 mL, 0.307 mmol, 0.2 equiv.). The mixture was stirred at 145 °C using an oil bath for 17 h under a nitrogen atmosphere. The reaction mixture was allowed to cool before being poured onto H_2O (40 mL) and extracted with DCM (3 × 50 mL). The combined organic layers were dried over $MgSO_4$ and the organic solvent was removed under reduced pressure. The crude product was purified by washing with acetone to obtain a white solid which was a mix of the tCz-IPm and tCz-BrPm. The product was used for the next step without further purification.

Step 3: synthesis of bis(2-(3,6-di-*tert*-butyl-9H-carbazol-9-yl) pyrimidin-5-yl) sulfane (tCz-PmS).

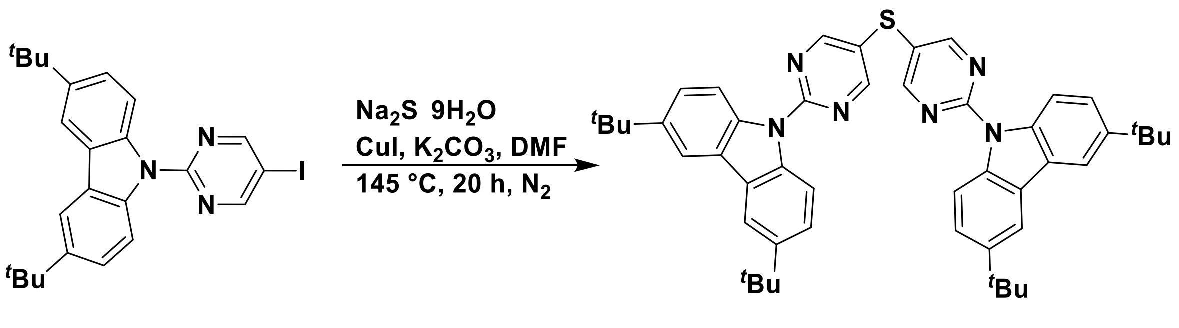

Figure **S3**. Reaction scheme for the synthesis of tCz-PmS.

To an oven dried flask were added tCz-Ipm (1.38 g, 2.86 mmol, 1 equiv.), sodium sulfide nonahydrate (0.247 g, 1.03 mmol, 0.6 equiv.) CuI (0.033 g, 0.28 mmol, 0.1 equiv.) and K_2CO_3 (0.710 g, 5.14 mmol, 3 equiv.). The flask was degassed by three cycles of vacuum-nitrogen purging and 20 mL of dry DMF was injected. The mixture was stirred at 145 °C using an oil bath for 20 h under a nitrogen atmosphere. The reaction mixture was allowed to cool before

being poured into 75 mL of icy water and extracted with ethyl acetate (3 × 40 mL). The combined organic layers were dried over $MgSO_4$ and the organic solvent was removed under reduced pressure. The crude product was purified by silica gel column chromatography. DCM:Hexane=1:1 was used as eluent to afford tCz-PmS as a white solid. **Yield:** 746 mg, 35%. **Rf:** 0.52 (33% DCM:Hexane). **^{1}H NMR (400 MHz, $CDCl_3$), δ (ppm):** 8.87 (s, 4H), 8.75 (dd, 4H), 8.04 (d, 4H), 7.53 (dd, 4H), 1.46 (s, 36H). The ^{1}H NMR spectrum is consistent with the literature.[28]

Step 4: synthesis of 9,9'-(sulfonylbis(pyrimidine-5,2-diyl))bis(3,6-di-*tert*-butyl-9H-carbazole) (**pDTCz-DPmS**) was completed according to literature procedure.[28]

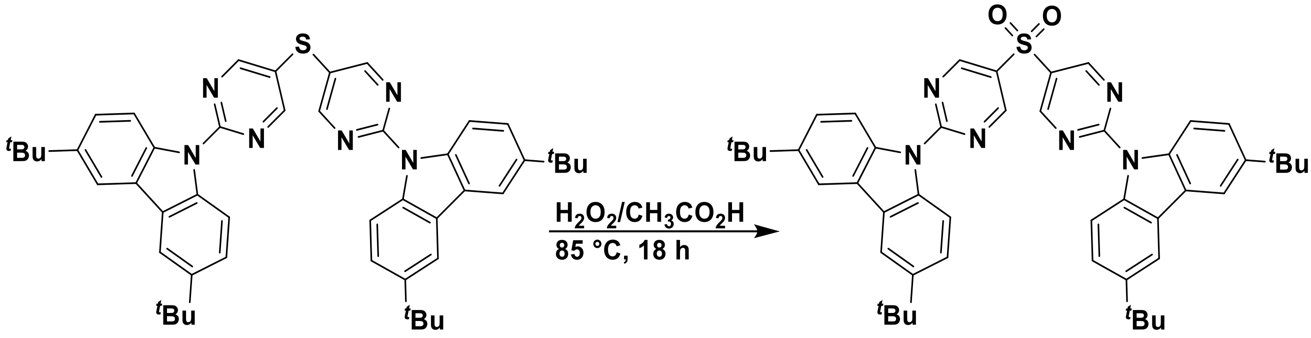

Figure **S4**. Reaction scheme for the synthesis of **pDTCz-DPmS**.

To a flask were added tCz-PmS (0.302 g, 0.41 mmol, 1 equiv.) and acetic acid (8 mL). To this suspension was added H_2O_2 (30 wt%, 12 mL, 1 equiv.) and mixture was heated to 85 °C using an oil bath for 18 h. The mixture was then poured into 40 mL of icy water and extracted with DCM (3 × 25 mL). The combined organic layers were dried over $MgSO_4$, filtered and the organic solvent was removed under reduced pressure. The crude product was purified by silica gel column chromatography. DCM:Hexanes = 4:1 was used as the eluent to afford **pDTCz-DPmS** as a white solid. The product was further purified by recrystallization in DCM:Hexanes. **Yield:** 124 mg, 39%. **Rf:** 0.68 (75% DCM:Hexane). **Mp:** 292-294 °C. **Lit.:** 292-294 °C.[28] **^{1}H NMR (400 MHz, $CDCl_3$) δ (ppm):** 9.31 (s, 4H), 8.84 (d, J = 8.9 Hz, 4H), 8.04 (d, J = 2.0 Hz, 4H), 7.57 (dd, J = 8.9, 2.0 Hz, 4H), 1.48 (s, 36H). The ^{1}H NMR spectrum is consistent with that in the literature.[28]

Electrochemistry

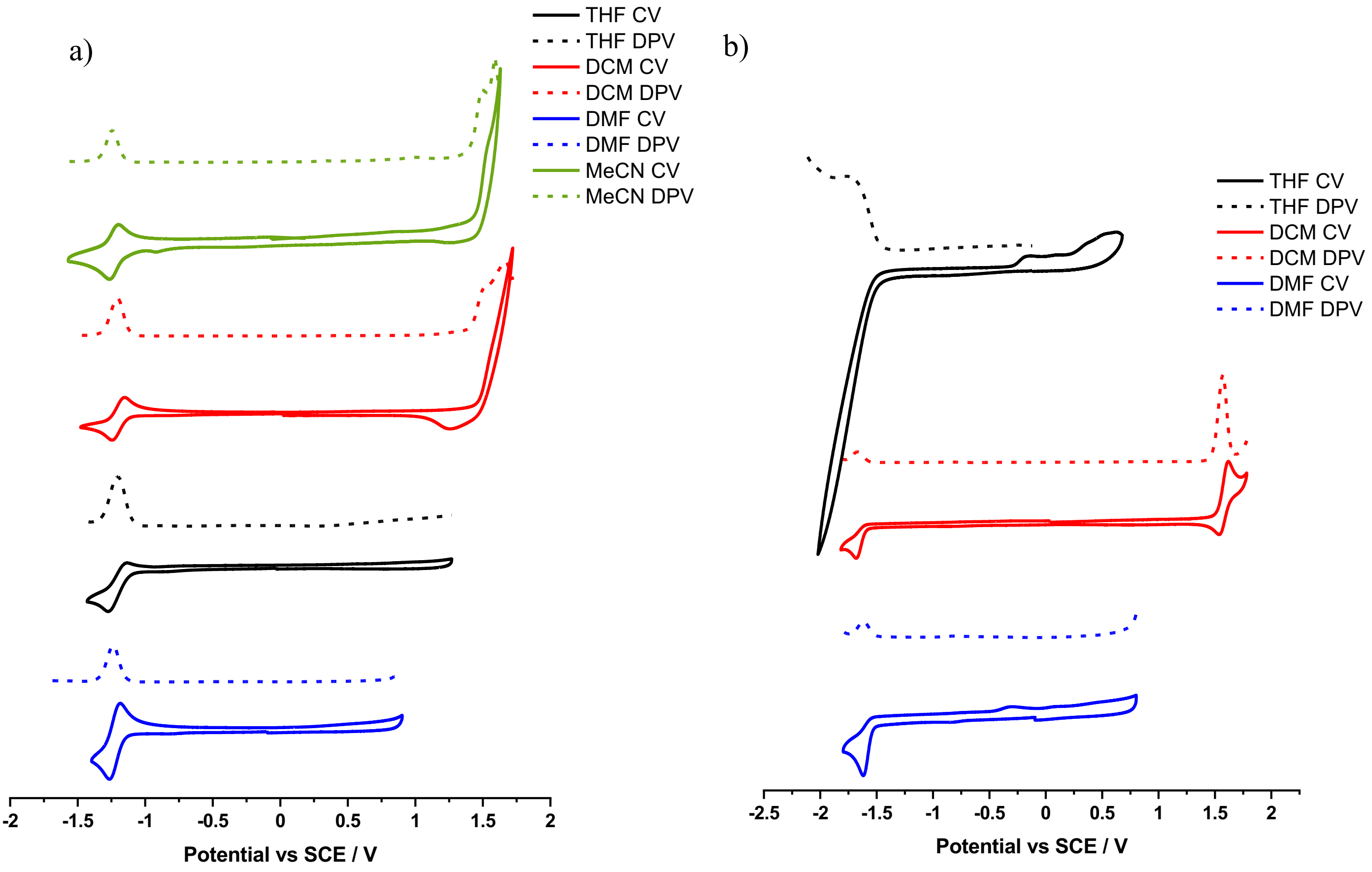

Figure **S5**. CVs and DPVs of a) **4CzIPN** and b) **pDTCz-DPmS** in a range of solvents, reported vs SCE at scan rate of 0.1 V s^{-1}.

Table **S1**. Redox potentials and optical gaps for **4CzIPN** and **pDTCz-DPmS**.[a]

PC		THF	DCM	DMF	MeCN
**4CzIPN**	E_{ox} / V		1.51		1.50
	E_{red} / V	-1.20	-1.21	-1.24	-1.24
	E^{*}_{ox} / V		-1.09		-1.15
	E^{*}_{red} / V	1.46	1.39	1.40	1.41
	$E_{0,0}$ / eV	2.66	2.60	2.64	2.65
**pDTCz-DPmS**	E_{ox} / V		1.57		
	E_{red} / V	-1.77	-1.67	-1.62	
	E^{*}_{ox} / V		-1.44		
	E^{*}_{red} / V	1.32	1.34	1.48	
	$E_{0,0}$ / eV	3.09	3.01	3.10	

[a]All redox potentials are reported vs SCE. E_{ox} and E_{red} values obtained from DPV max and $E^{*}_{ox} = E_{ox} - E_{0,0}$ and $E^{*}_{red} = E_{red} + E_{0,0}$. $E_{0,0}$ obtained from the intersection point between the normalized absorption and emission spectra.

Photophysical measurements

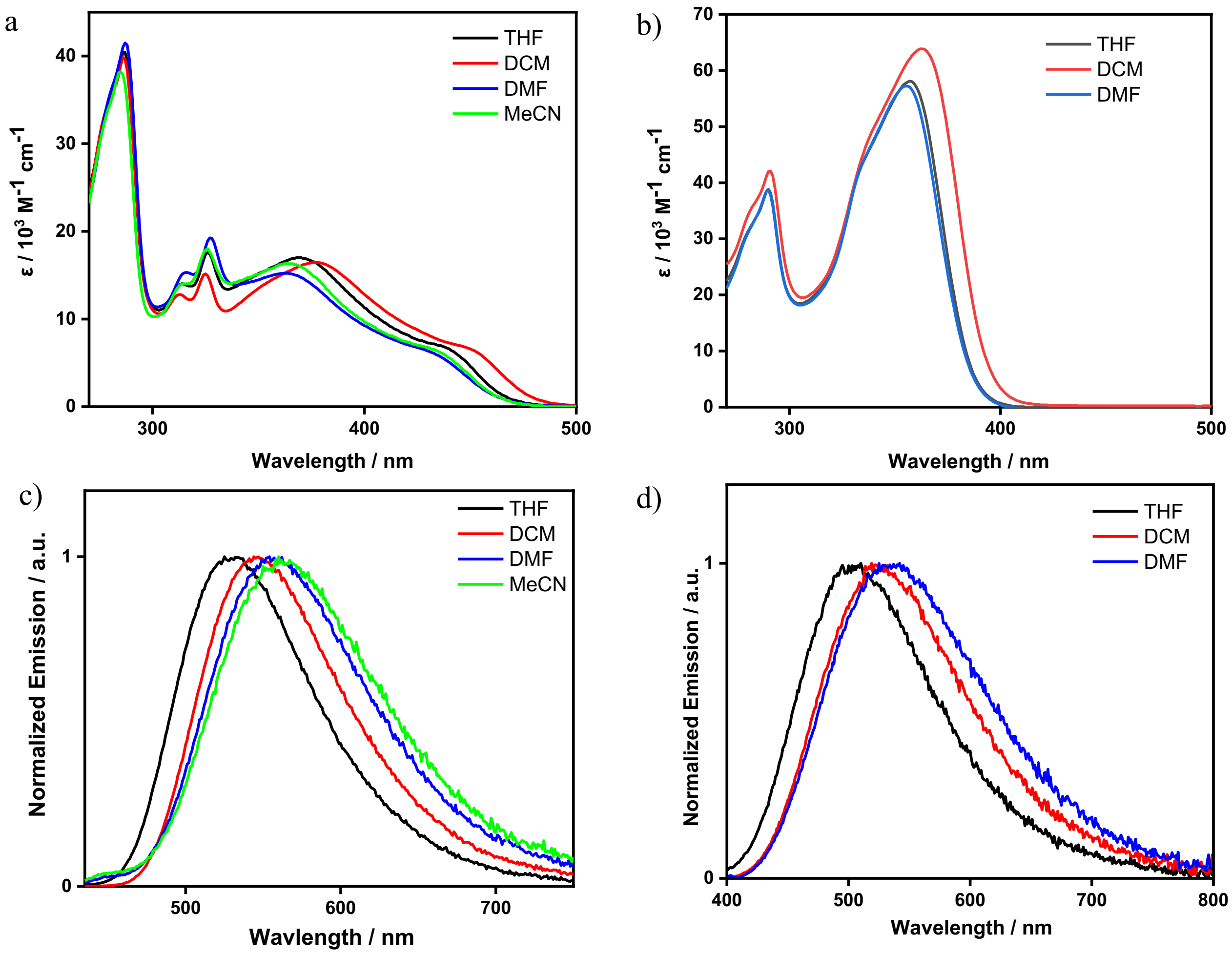

Figure **S6**. Solvatochromic absorption study for a) **4CzIPN** and b) **pDTCz-DPmS** and solvatochromic PL study of c) **4CzIPN** and d) **pDTCz-DPmS**. λexc = 420 nm for **4CzIPN** and 360 nm for **pDTCz-DPmS**. Measurements performed at room temperature under air.

Table **S2**. Absorption and emission maxima of **4CzIPN** and **pDTCz-DPmS** in different solvents.

Solvent	λ_{abs} / nm (ε / 10^3 M^{-1} cm^{-1})		λ_{PL}/ nm		$E_{0,0}$ / eV	
	4CzIPN	pDTCz-DPmS	4CzIPN	pDTCz-DPmS	4CzIPN	pDTCz-DPmS
Toluene	441 (6)	365 (59)	507	480	2.59	3.12
THF	438 (8)	357 (58)	525	505	2.66	3.09
DCM	448 (7)	363 (64)	544	524	2.60	3.01
DMF	428 (6)	355 (57)	554	535	2.64	3.10
MeCN	432 (6)		560	546	2.65	

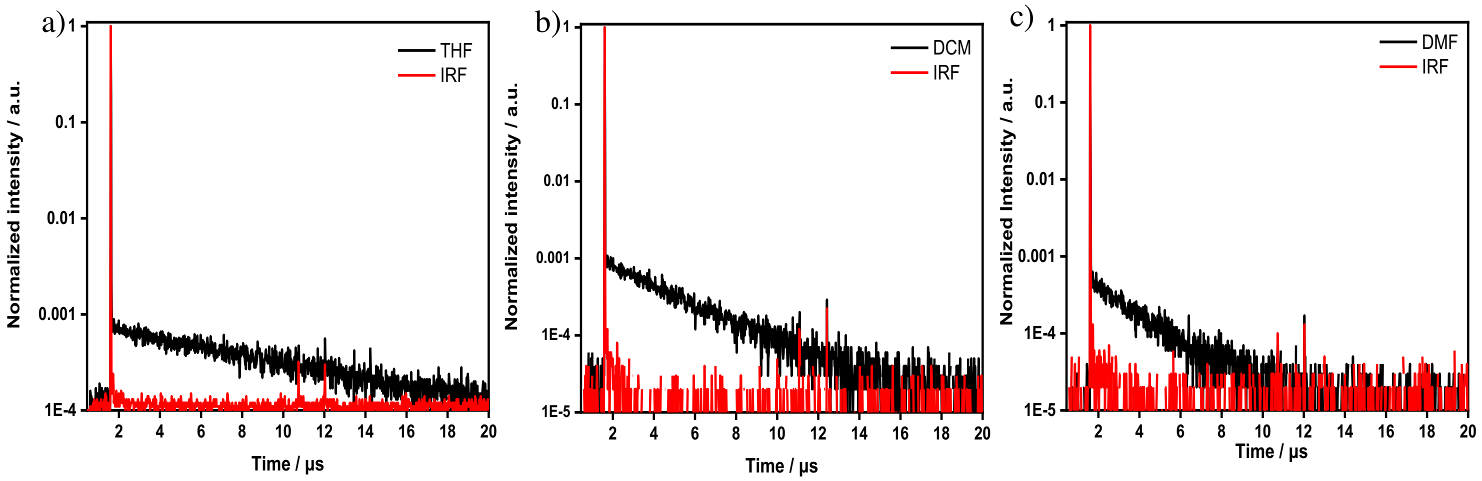

Figure **S7**. Time resolved PL decay of **pDTCz-DPmS** recorded in a) THF, b) DCM and c) DMF under vacuum in 10^{-5} M solutions with λ_{exc} = 378 nm.

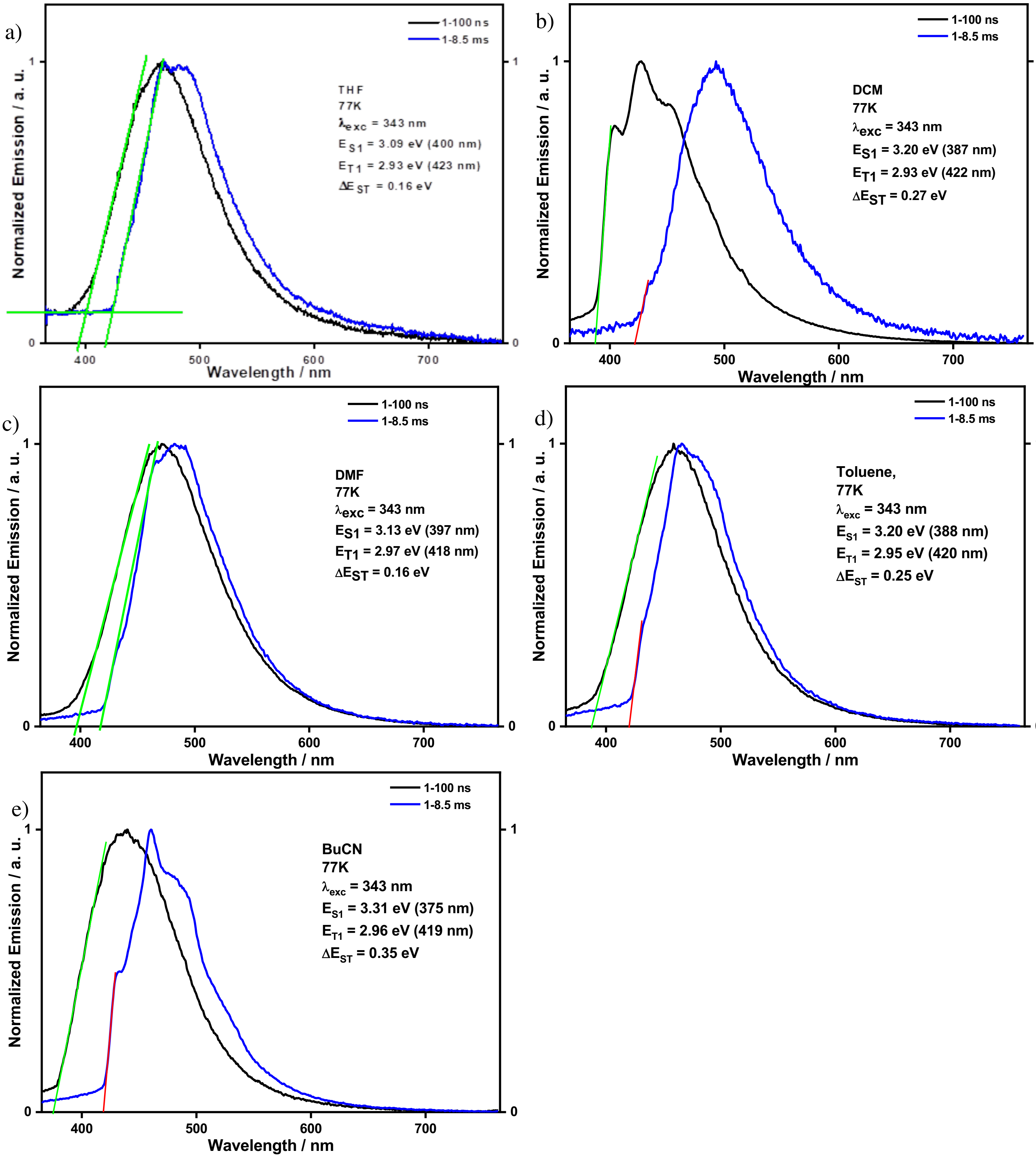

Figure **S8**. Prompt fluorescence and phosphorescence spectra of **pDTCz-DPmS** in a) THF, b) DCM, c) DMF, d) toluene and e) BuCN measured in 10^{-5} M solutions at 77 K. In all cases, λ_{exc} = 343 nm, and prompt and delayed fluorescence spectra were obtained in the 1–100 ns and 1–8.5 ms time range, respectively

Mechanistic studies of the decarboxylative addition reaction of N-Cbz-Pro to diethyl maleate

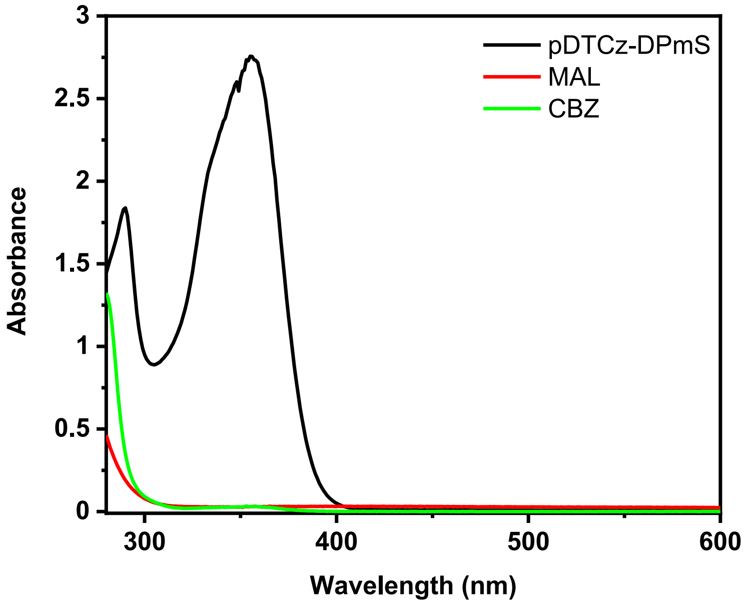

Figure **S9**. Absorption spectra of **pDTCz-DPmS** (black line, 4 × 10^{-5} M), diethylmaleate (red line, 5 × 10^{-4} M) and *N*-Cbz-Pro (green line, 5 × 10^{-4} M) in DMF.

Figure **S9** demonstrates that the photocatalyst **pDTCz-DPmS** is the only species absorbing the 390 nm LED light in the decarboxylative addition of *N*-Cbz-Pro to diethyl maleate.

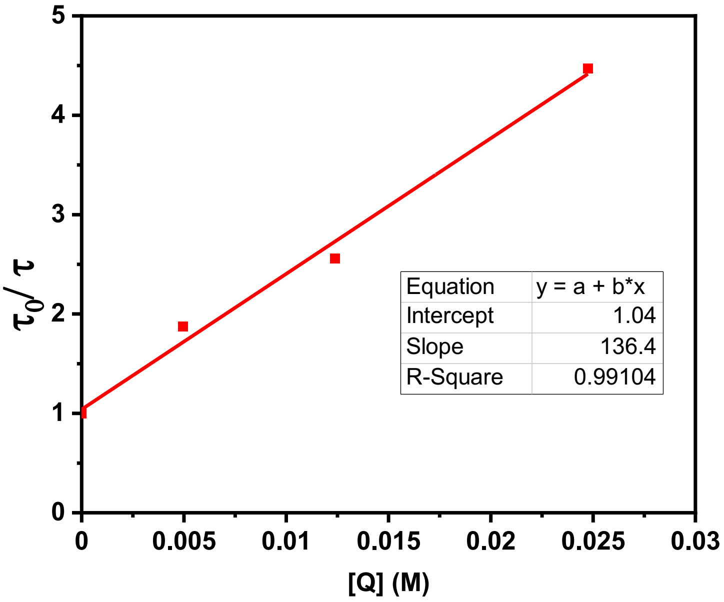

Figure **S10**. Stern-Volmer plot of the quenching of the TADF emission of **pDTCz-DPmS** in DMF by sequential addition of diethylmaleate.

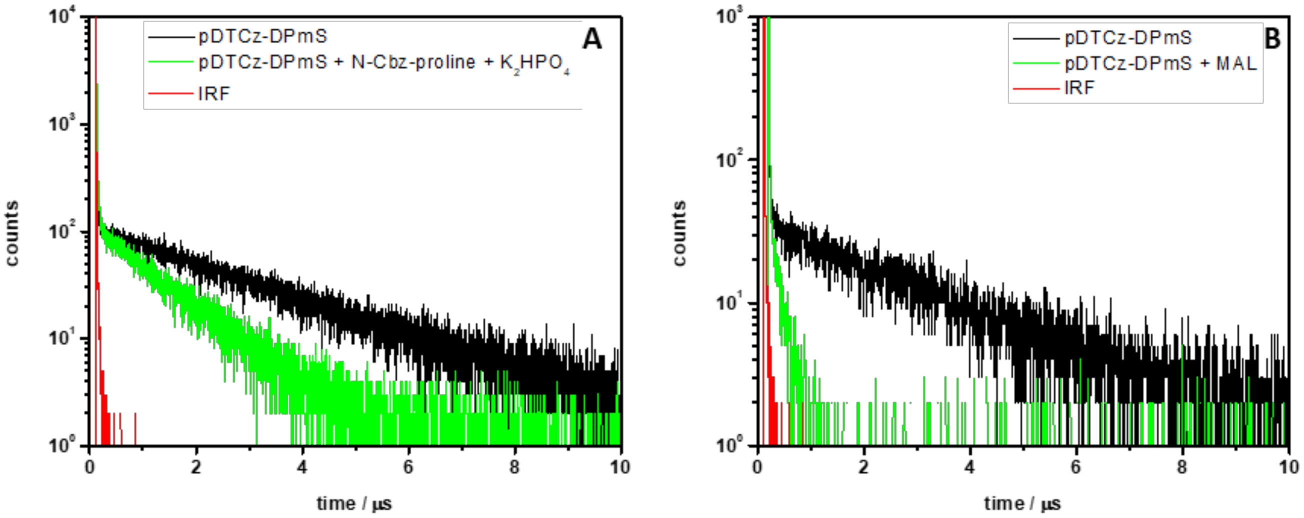

Figure **S11**. Time resolved PL decay of a deaerated solution of **pDTCz-DPmS** (4.27×10^{-5} M, black line) in DMF and after addition of: a) *N*-Cbz-Pro (0.05 M) and K_2HPO_4 (saturated solution) and b) diethyl maleate (0.055 M) with λ_{exc} = 340 nm.

The quenching process is studied following the changes in the lifetime of the delayed emission of the photocatalyst by addition of increasing amounts of diethylmaleate or *N*-Cbz-Pro. The photocatalyst is not quenched by the protonated form of *N*-Cbz-Pro; deprotonation of the carboxylic acid is required. The quenching of the deprotonated form of *N*-Cbz-Pro is evaluated after the addition of *N*-Cbz-Pro and K_2HPO_4 and 12 hours of stirring under inert atmosphere.

To evaluate the quenching of **pDTCz-DPmS** under the reaction conditions, we evaluated the quenching efficiency of species *i* according to the following formula:

$$\eta^i = \frac{k_q^i \cdot [Q]^i}{k_{nr} + k_r + \sum_0^n k_q^i \cdot [Q]^i} \cdot 100$$

where $k_{nr} + k_r = \frac{1}{\tau 0}$ are the intramolecular deactivation pathways and k_q^i is the quenching constant of the quencher species *i*.

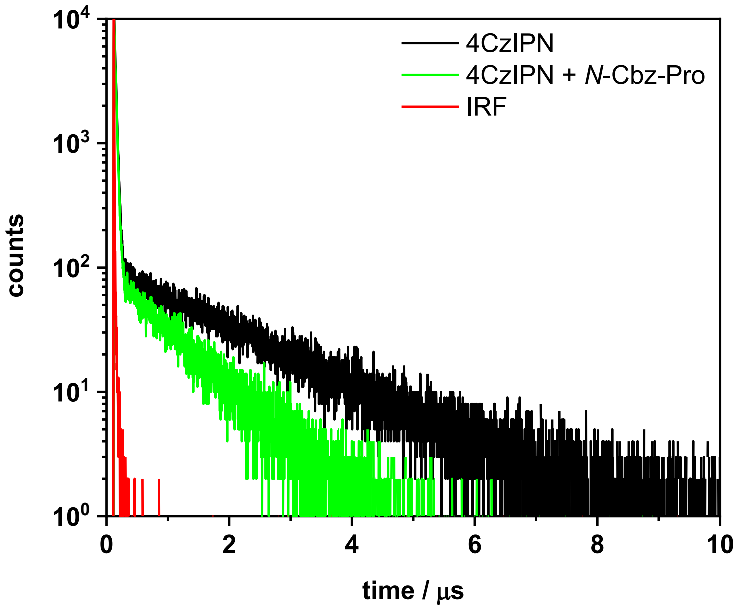

Figure **S12**. Time resolved PL decay of a deaerated solution of **4CzIPN** (5.6×10^{-5} M, black line) in DMF and after addition of *N*-Cbz-Pro (0.05 M, red line) and K_2HPO_4 (saturated solution) with λ_{exc} = 340 nm.

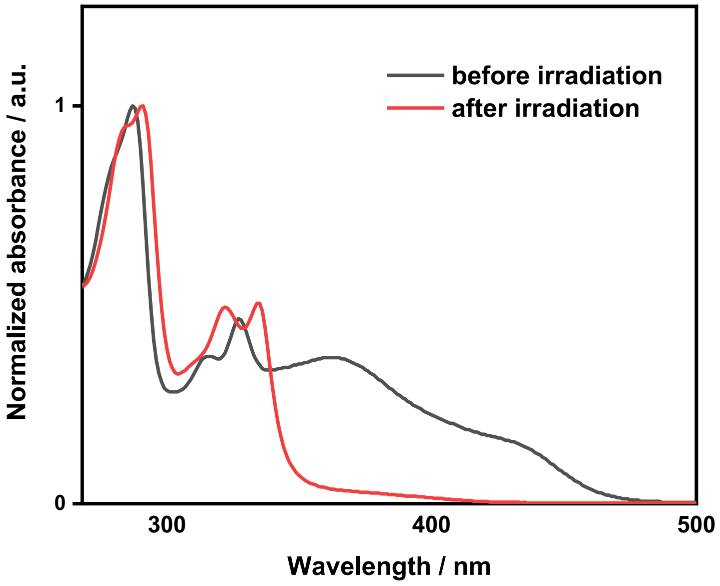

Figure **S13**. UV-Vis absorption spectra of **4CzIPN**, N-Cbz-Pro and Cs_2CO_3 (1:4:4 equiv.) in DMF before and after 30 minutes of irradiation under N_2 with 390 nm Kessil lamp.

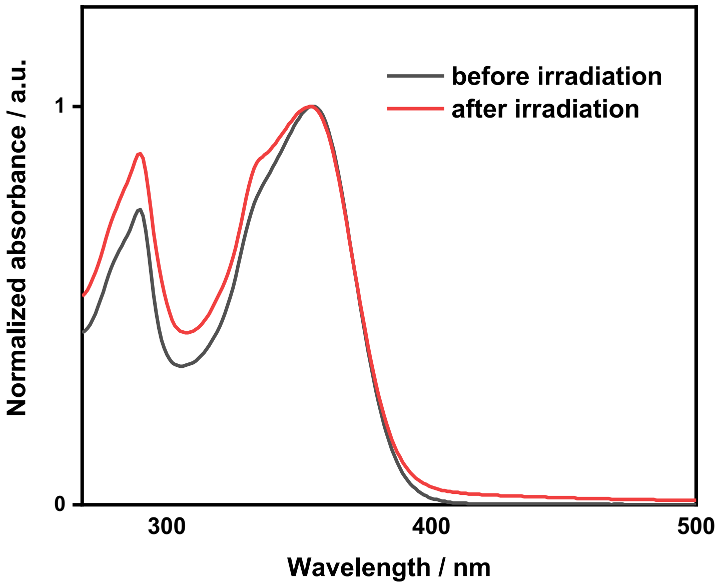

Figure **S14**. UV-Vis absorption spectra of **pDTCz-DPmS,** N-Cbz-Pro and Cs_2CO_3 (1:4:4 equiv.) in DMF before and after 30 minutes of irradiation under N_2 with 390 nm Kessil lamp.

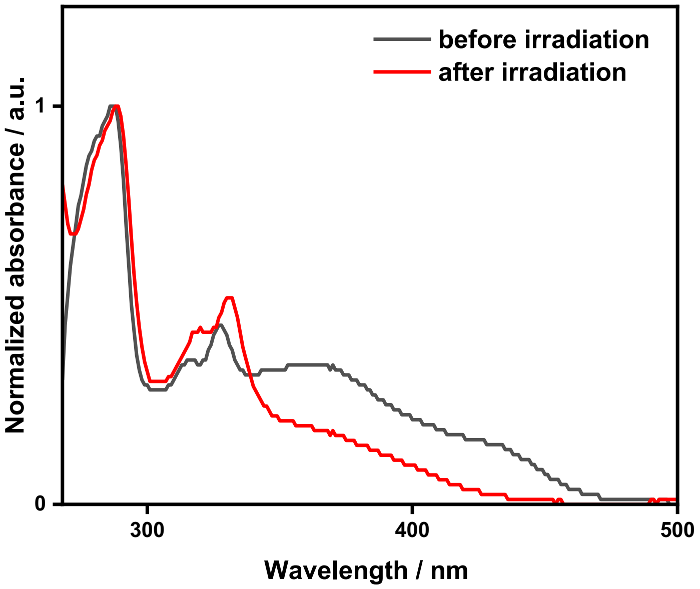

Figure **S15**. UV-Vis absorption spectra of **4CzIPN**, N-Cbz-Pro, K_2HPO_4 and diethyl maleate (0.02 : 1 : 1.1 : 1.1 ratio as in the reaction) in DMF before and after 24 minutes of irradiation under N_2 with 390 nm Kessil lamp.

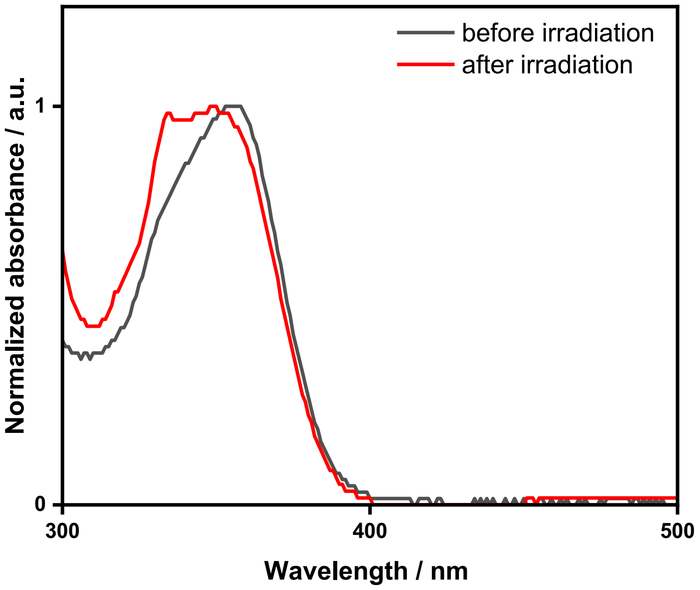

Figure **S16**. UV-Vis absorption spectra of **pDTCz-DPmS**, N-Cbz-Pro, K_2HPO_4 and diethyl maleate (0.02 : 1 : 1.1 : 1.1 ratio as in the reaction) in DMF before and after 24 minutes of irradiation under N_2 with 390 nm Kessil lamp.

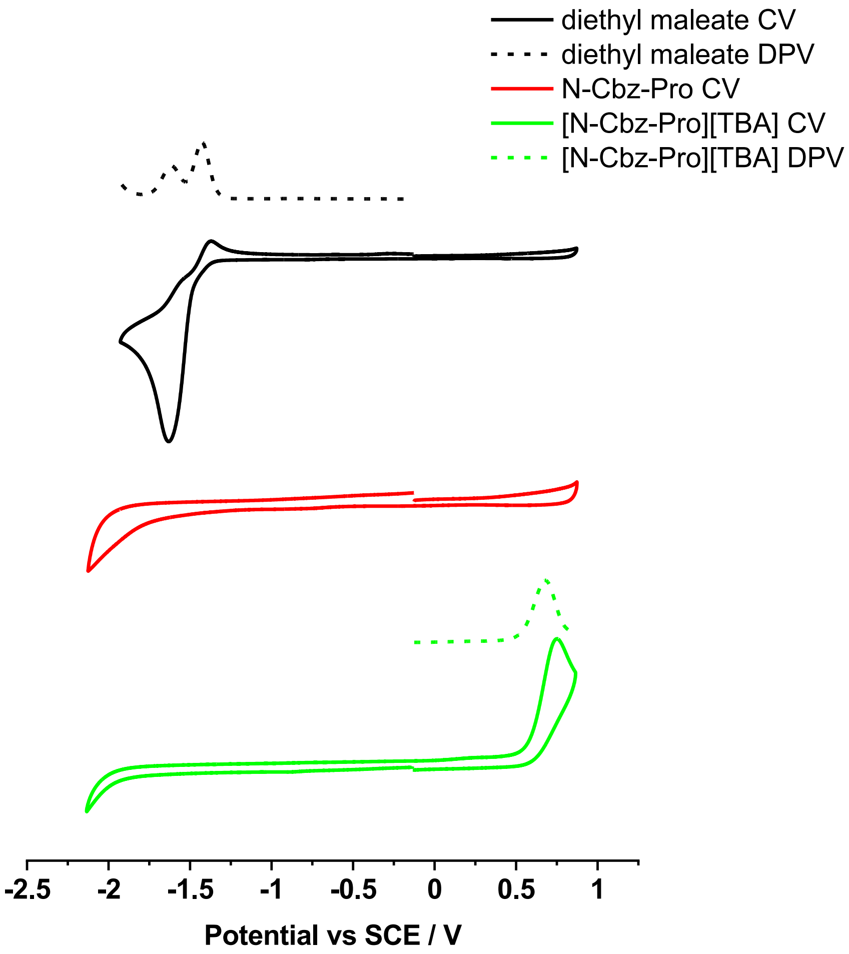

Figure **S17**. CV and DPV of diethyl maleate (black), *N*-Cbz-Pro (red) and *tert*-butylammonium *N*-Cbz-Pro salt (green) all in DMF, reported vs SCE at scan rate of 0.1 V s^{-1}. Due to the absence of electrochemical activity in the window scanned, only the CV was obtained for *N*-Cbz-Pro. CV and DPV of [TBA][N-Cbz-Pro] was obtained according to the procedure outlined in reference [29].

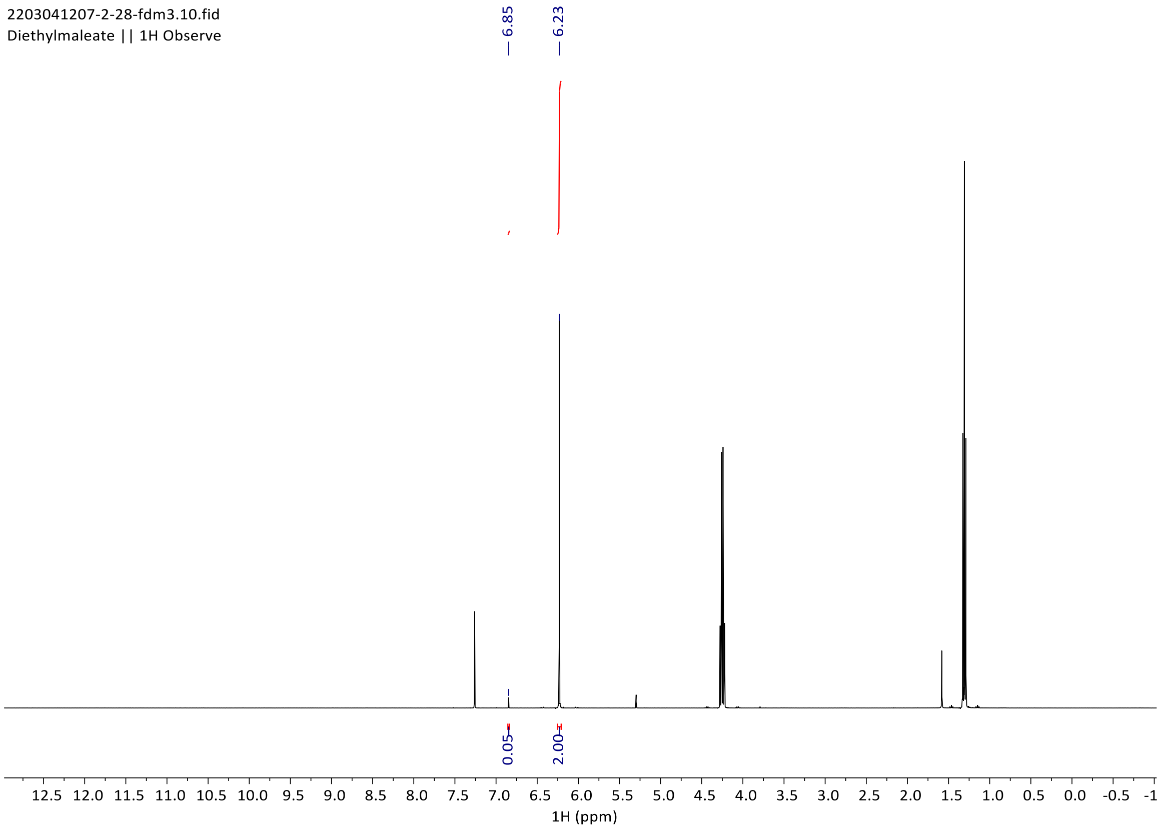

Figure **S18.** ^{1}H NMR of diethyl maleate reagent with integration of the relevant peaks corresponding to the *Z* maleate isomer (6.23 ppm) and the *E* fumarate isomer (6.85 ppm) in $CDCl_3$.

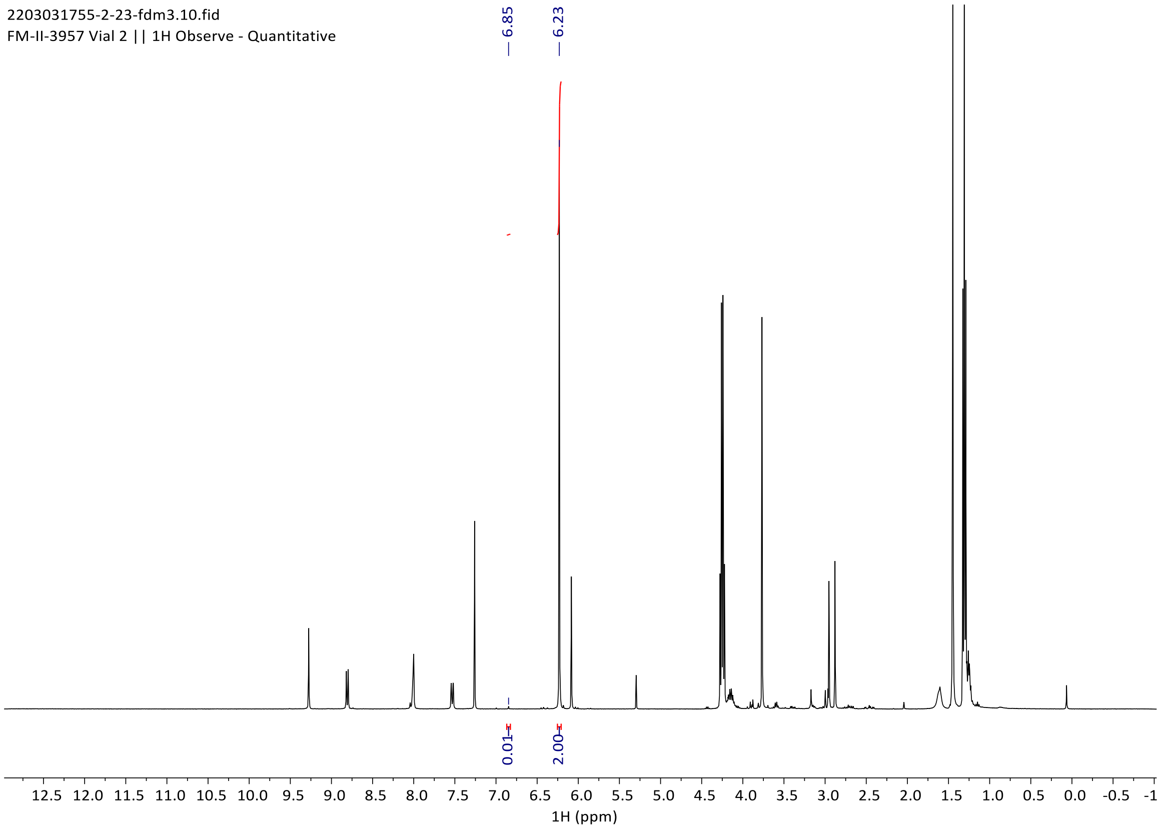

Figure **S19**. ^{1}H NMR obtained in $CDCl_3$ after irradiation of diethyl maleate in the presence of **pDTCz-DPmS**, according to the reaction conditions and concentrations outlined for the decarboxylative addition of *N*-Cbz-Pro to diethyl maleate, but in the absence of *N*-Cbz-Pro and K_2HPO_4. The integrated peaks shown correspond to the *Z* maleate isomer (6.23 ppm) and the *E* fumarate isomer (6.85 ppm).

From the ^{1}H NMR data shown in Figures **S18** and **S19**, we can conclude that no *Z* → *E* isomerisation of diethyl maleate is taking place upon irradiation with **pDTCz-DPmS**.

Photocatalysis

Photocatalysis experiments were conducted using a custom-built photoreactor, as shown in Figure **S20**, allowing for up to 8 parallel photochemical reactions (7 mL) at a time. The photochemistry reaction chamber is filled with mirrors to evenly distribute light. The reactor is placed upon a magnetic stirrer plate allowing for reactions to be completed with stirring. Reactions are irradiated using Kessil PR160 LED sources. For Kessil PR160-390 nm, the chosen LED source for photocatalysis reactions completed in this study, the power consumption maximum is 52 W, with the average intensity measured from 1 cm distance being 352 mW cm^{-2}. The intensity on each lamp is tuneable, with the maximum intensity selected for all photocatalytic reactions. A cooling fan is directed at the photoreactor to ensure the reaction mixture maintains at room temperature, which is further guaranteed by the presence of two fans on the photoreactor itself.

After the photoreactions were completed, the products were analysed by ^{1}H NMR spectroscopy with an internal standard, either 1,3,5-trimethoxybenzene or 1,4-(bis(trimethylsilyl)benzene). All yields shown represent the mean yield from at least two reactions with the associated standard deviation.

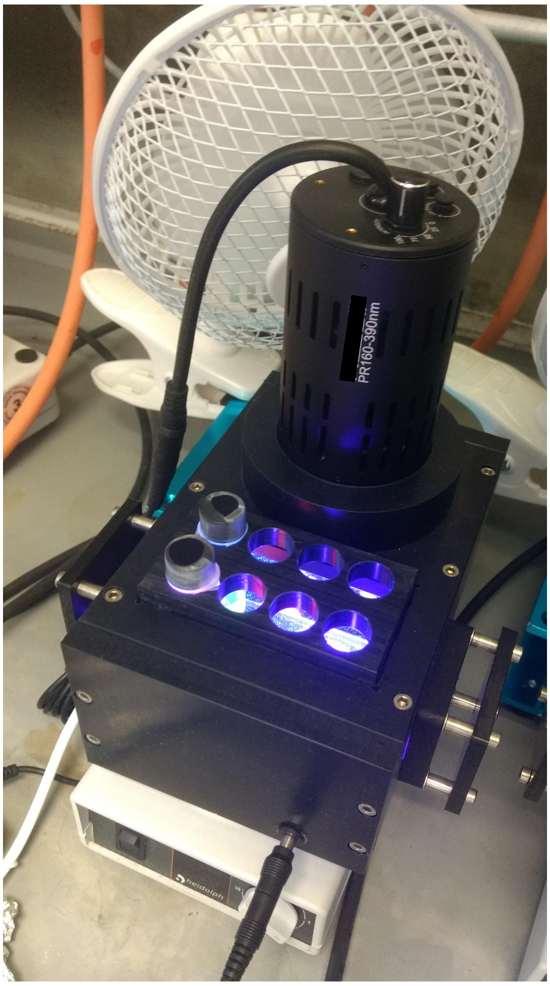

Figure **S20**. Experimental setup for photocatalysis reactions.

Procedure for oxidative quenching reaction:

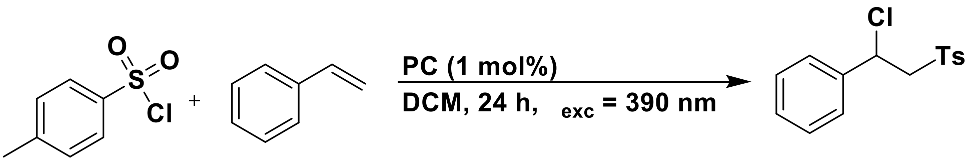

Figure **S21**. Reaction scheme for the oxidative quenching reaction.

To an oven-dried vial was added *p*-toluenesulfonyl chloride (48 mg, 0.25 mmol, 1 equiv.) styrene (0.017 mL, 0.25 mmol, 1 equiv.) and photocatalyst (1 mol%, 0.0025 mmol). The vial was purged with N_2 for 5 min and dry DCM (1.0 mL) was added before further N_2 purging for 10 min. The solution was stirred at room temperature while being irradiated by Kessil lamp (λ_{exc} = 390 nm) for 24 hours. After removal of solvent, the crude product was purified by flash column chromatography (5:1 hexane:EtOAc) to afford the final product as a white solid. **R_f:** 0.30 (5:1 hexane:EtOAc). **^{1}H NMR (400 MHz, $CDCl_3$), δ (ppm):** 7.63 (d, 2H), 7.29 – 7.22 (m, 7H), 5.33 (t, 1H), 3.94 (dd, 1H), 3.85 (dd, 1H), 2.41 (s, 3H). The ^{1}H NMR spectrum is consistent with that in the literature.[30]

Table **S3**. ^{1}H NMR yields obtained from the oxidative quench reaction.[a]

Photocatalyst	λ_{exc} / nm	Solvent	^{1}H NMR yield / %
None	390	MeCN	0
$[Ru(bpy)_3](PF_6)_2$	456	MeCN	81 ± 1
$[Ru(bpy)_3](PF_6)_2$	390	MeCN	42 ± 1
$[Ru(bpy)_3](PF_6)_2$	390	DCM	64 ± 3
4CzIPN	390	DCM	10 ± 1
pDTCz-DPmS	390	DCM	16 ± 2

[a] Reaction conditions as stated in the procedure above unless otherwise noted.

Procedure for reductive quenching reaction:

1) Pinacol coupling

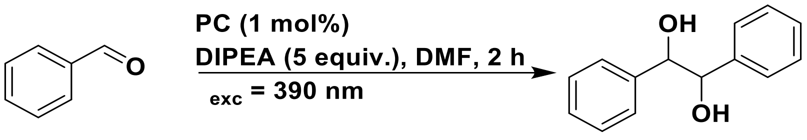

Figure **S22**. Reaction scheme for the pinacol coupling.

To an oven-dried vial was added benzaldehyde (0.020 mL, 0.2 mmol, 1 equiv.), DIPEA (0.174 mL, 1 mmol, 5 equiv.) and photocatalyst (1 mol%, 0.002 mmol). The vial was purged with N_2 for 5 min and dry DMF (2.0 mL) was added before further N_2 purging for 10 min. The solution

was stirred at room temperature while being irradiated by Kessil lamp (λ_{exc} = 390 nm) for 2 or 24 hours. After removal of solvent, the crude product was purified by flash column chromatography (1:5 EtOAc:Hexane → 100% EtOAc) to afford the product as a white solid. **R_f:** 0.83 (100% EtOAc). **^{1}H NMR (400 MHz, $CDCl_3$) of meso and dl, δ (ppm):** 7.33 – 7.27 (m, 6H), 7.26 – 7.21 (m, 10H), 7.12 (dd, J = 6.5, 2.9 Hz, 4H, dl), 4.81 (s, 2H, meso), 4.66 (s, 2H, dl), 3.17 (br s, 2H, dl), 2.50 (br s, 2H, meso). The ^{1}H NMR spectrum is consistent with that in the literature.[31]

Table **S4**. ^{1}H NMR yields obtained from the pinacol reaction.[a]

Photocatalyst	Time / h	^{1}H NMR yield / %
None	2	18 ± 2
None	24	50 ± 2
$[Ir(ppy)_2(dtbbpy)]PF_6$	2	43 ± 3
$[Ir(ppy)_2(dtbbpy)]PF_6$	24	74 ± 3
4CzIPN	2	68 ± 0
4CzIPN	24	76 ± 3
pDTCz-DPmS	2	32 ± 1
pDTCz-DPmS	24	80 ± 3

[a] Reaction conditions as stated in the procedure above unless otherwise noted.

2) Decarboxylative addition of *N*-Cbz-Pro to diethyl maleate

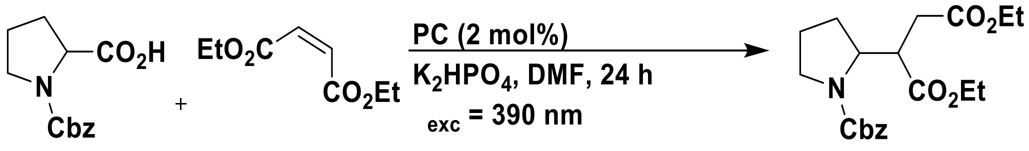

Figure **S23**. Reaction scheme for the decarboxylative addition of *N*-Cbz-Pro to diethyl maleate.

To an oven-dried vial was added *N*-Cbz-Pro (50 mg, 0.2 mmol, 1 equiv.), K_2HPO_4 (38 mg. 0.22 mmol, 1.1 equiv.), diethyl maleate (0.036 mL, 0.22 mmol, 1.1 equiv.) and photocatalyst (2 mol%, 0.004 mmol). The vial was purged with $N_2$for 5 min and dry DMF (4.0 mL) was added before further N_2 purging for 10 min. The solution was stirred at room temperature while being irradiated by Kessil lamp (λ_{exc} = 390 nm) for 24 hours. After irradiation, the mixture was poured into water and extracted with DCM (3×15 mL). The combined organic phases were dried over Na_2SO_4 and filtered. The solvent was removed under reduced pressure and the residue was purified by flash chromatography on silica gel (3-20% EtOAc:Hexane) to afford the product as a colourless solid. **R_f:** 0.19 (1:4 EtOAc:Hexane). **^{1}H NMR (400 MHz, $CDCl_3$), δ (ppm):** 7.49

– 7.31 (m, 5H), 5.30 – 5.03 (m, 2H), 4.38 – 4.28 (m, 1H), 4.24 – 4.02 (m, 4H), 3.77 – 3.46 (m, 2H), 3.44 – 3.20 (m, 1H), 2.88 – 2.66 (m, 1H), 2.57 – 2.24 (m, 1H), 2.01 – 1.72 (m, 4H), 1.32 – 1.16 (m, 6H). The ^{1}H NMR spectrum is consistent with that in the literature.[32]

Table **S5**. ^{1}H NMR yields obtained from the decarboxylative addition of *N*-Cbz-Pro to diethyl maleate.[a]

Photocatalyst	^{1}H NMR yield / %
None	0
$[Ir(dF(CF_3)ppy)_2(dtbbpy)]PF_6$	99 ± 0
4CzIPN	99 ± 0
pDTCz-DPmS	64 ± 3

[a] Reaction conditions as stated in the procedure above.

Procedure for *E/Z* isomerisation reaction:

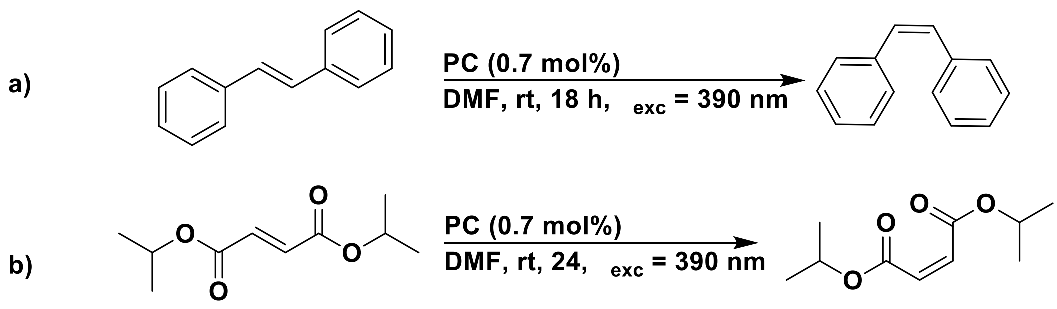

Figure **S24**. Reaction scheme for the *E*/*Z* isomerisation of a) *E*-stilbene and b) di*iso*propylfumarate.

To an oven-dried vial was added *E*-stilbene (36 mg, 0.2 mmol, 1 equiv.) or diisopropyl fumarate (0.039 mL, 0.2 mmol, 1 equiv.) and photocatalyst (0.7 mol%, 0.0014 mmol). The vial was purged with N_2 for 5 min and dry DMF (1.0 mL) was added before further N_2 purging for 10 min. The solution was stirred at room temperature while being irradiated by Kessil lamp (λ_{exc} = 390 nm) for 18 or 24 hours (for *E*-stilbene and di*iso*propyl fumarate, respectively). After removal of solvent, the crude product was purified by flash column chromatography.

Z-stilbene: white solid. **R_f:** 0.27 (*n*-pentane). **^{1}H NMR (400 MHz, $CDCl_3$), δ (ppm):** 7.29-7.16 (m, 10H), 6.61 (s, 2H). The ^{1}H NMR spectrum is consistent with that in the literature.[33]

Di*iso*propyl maleate: colourless oil. **R_f:** 0.20 (1:20 EtOAc:Petroleum ether). **^{1}H NMR (400 MHz, $CDCl_3$), δ (ppm):** 6.18 (s, 2 H), 5.11 (sept, 2H), 1.29 (d, 12 H). The ^{1}H NMR spectrum is consistent with that in the literature.[34]

Table **S6**. ^{1}H NMR yields obtained for the E/Z isomerisation of alkenes.[a]

Photocatalyst	Substrate	^{1}H NMR yield / %
None	*E*-Stilbene	5 ± 0
None	di*iso*propyl fumarate	Trace
$[Ru(bpy)_3](PF_6)_2$	*E*-Stilbene	81 ± 1
$[Ir(dF(CF_3)ppy)_2(dtbbpy)]PF_6$	di*iso*propyl fumarate	58 ± 1
4CzIPN	*E*-Stilbene	87 ± 1
4CzIPN	di*iso*propyl fumarate	6 ± 1
pDTCz-DPmS	*E*-Stilbene	63 ± 4
pDTCz-DPmS	di*iso*propyl fumarate	81 ± 2

[a] Reaction conditions as stated in the procedure above unless otherwise noted.

Procedure for dual Ni(II) cross-coupling reaction:

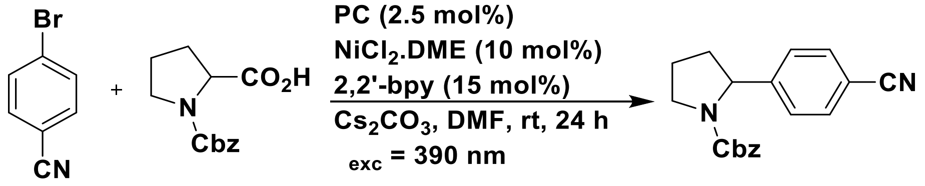

Figure **S25**. Reaction scheme for the dual Ni(II) cross-coupling reaction.

To an oven-dried vial was added *p*-bromobenzonitrile (36 mg, 0.2 mmol, 1 equiv.), *N*-Cbz-proline (74 mg, 0.3 mmol, 1.5 equiv.), $NiCl_2$.DME (4.4 mg, 20 µmol, 10 mol%), 2,2'-bipyridine (4.7 mg, 30 µmol, 15 mol%), Cs_2CO_3 (98 mg, 0.3 mmol, 1.5 equiv.) and photocatalyst (2.5 mol%, 0.005 mmol). The vial was purged with N_2 for 5 mins before the additions of dry DMF (5.0 mL) and a further 10 min of N_2 purging. The reaction mixture and stirred and irradiated with a Kessil lamp (λ_{exc} = 390 nm) for 24 hours. Upon completion, the mixture was added to H_2O (10 mL) and extracted with EtOAc (3 x 15 mL). The combined organic layers were dried over $MgSO_4$, filtered and the solvent removed *in vacuo*. The crude product was purified by flash column chromatography (15:85 EtOAc:Hexane) to afford the product as a foam. **R_f:** 0.14 (15:85 EtOAc:Hexane). **^{1}H NMR (400 MHz, $CDCl_3$), δ (ppm):** 7.57 (dd, 2H), 7.37 – 7.17 (m, 6H), 6.89 (d, 1H), 5.16 – 4.99 (m, 2H), 4.94 – 4.87 (m, 1H), 3.72 – 3.64 (m, 2H), 2.43 – 2.31 (m, 1H), 1.94 – 1.78 (m, 3H). ^{1}H The ^{1}H NMR spectrum is consistent with that in the literature.[35]

Table **S7**. ^{1}H NMR yields for the dual Ni(II) cross-coupling reaction.[a]

Photocatalyst	^{1}H NMR yield / %
None	24 ± 0
4CzIPN	99 ± 1
pDTCz-DPmS	72 ± 4

[a] Reaction conditions as stated in the procedure above.

NMR

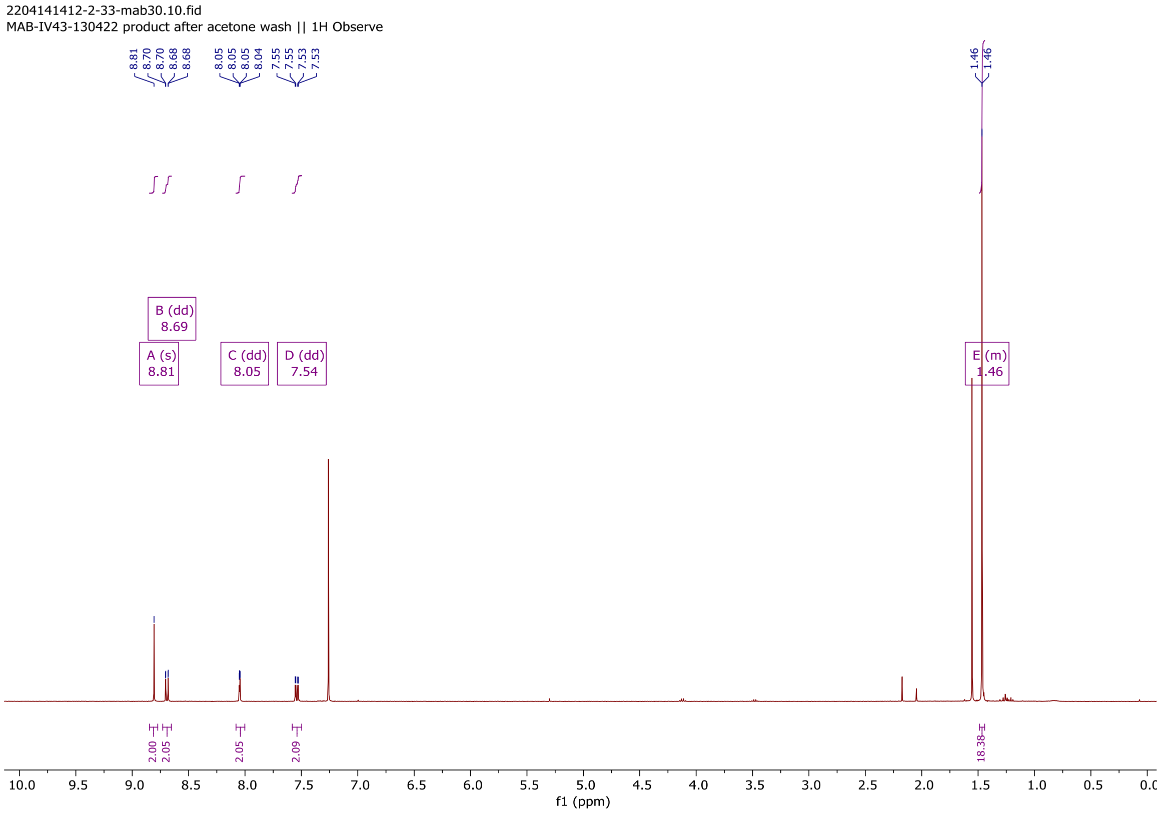

Figure **S26**. ^{1}H NMR spectrum of 9-(5-bromopyrimidin-2-yl)-3,6-di-*tert*-butyl-9H-carbazole (tCz-BrPm) in $CDCl_3$ at 400 MHz.

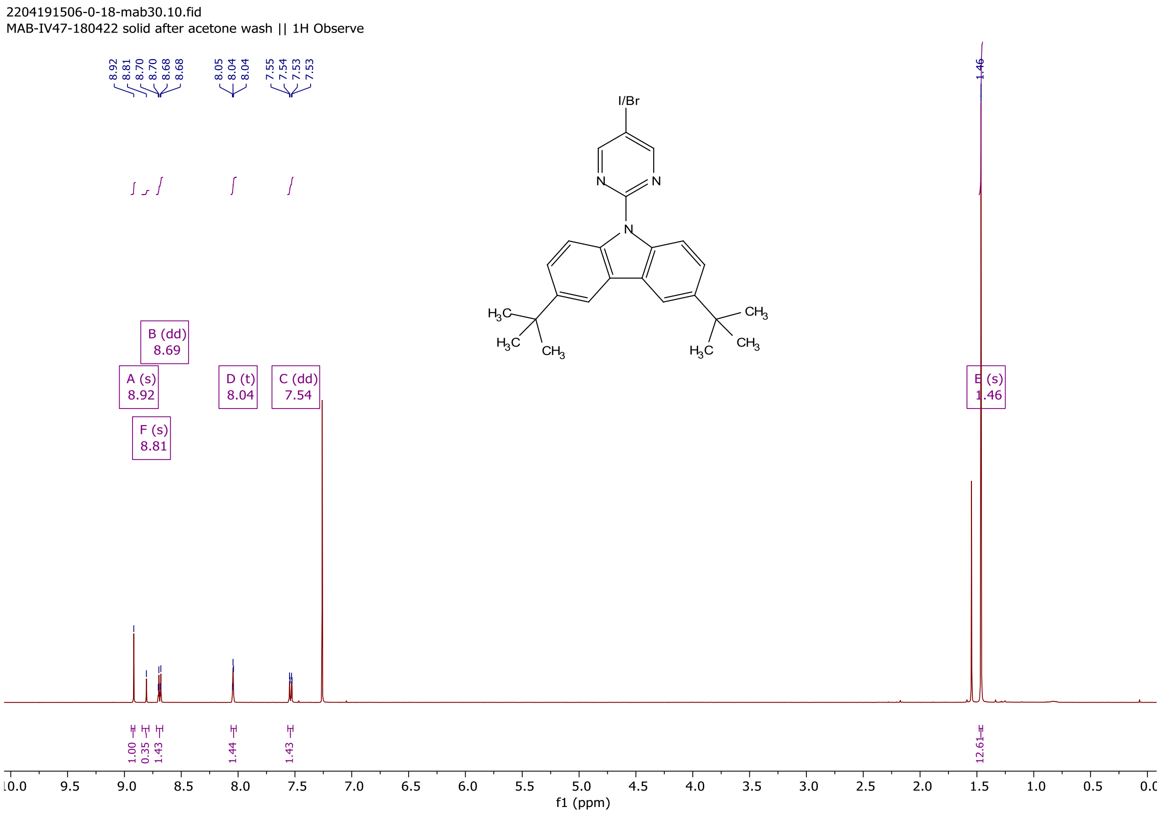

Figure **S27**. ^{1}H NMR spectrum of the mixture of 9-(5-bromopyrimidin-2-yl)-3,6-di-*tert*-butyl-9H-carbazole (tCz-BrPm) and 9-(5-iodoopyrimidin-2-yl)-3,6-di-*tert*-butyl-9H-carbazole (tCz-IPm) in $CDCl_3$ at 400 MHz.

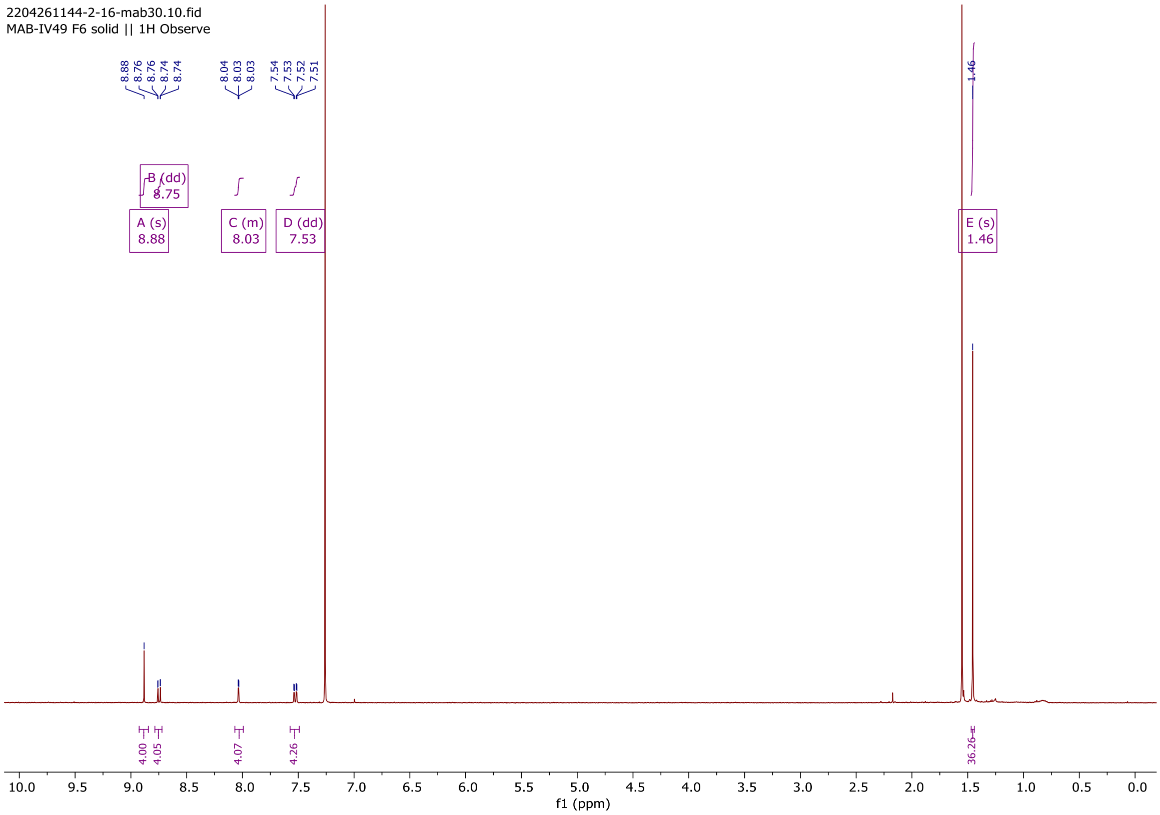

Figure **S28**. ^{1}H NMR spectrum of bis(2-(3,6-di-*tert*-butyl-9H-carbazol-9-yl)pyrimidin-5-yl)sulfane (tCz-PmS) in $CDCl_3$ at 400 MHz.

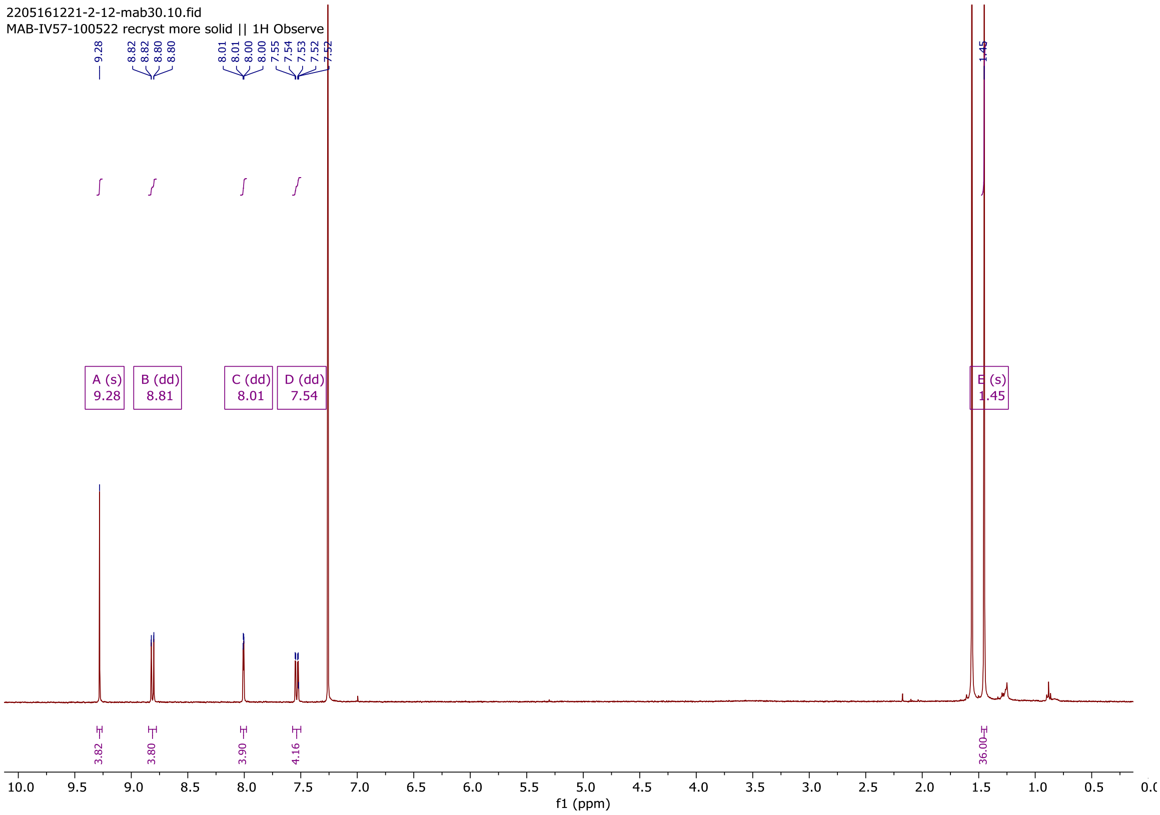

Figure **S29**. ^{1}H NMR spectrum of 9,9'-(sulfonylbis(pyrimidine-5,2-diyl))bis(3,6-di-*tert*-butyl-9H-carbazole) (**pDTCz-DPmS**) in $CDCl_3$ at 400 MHz.

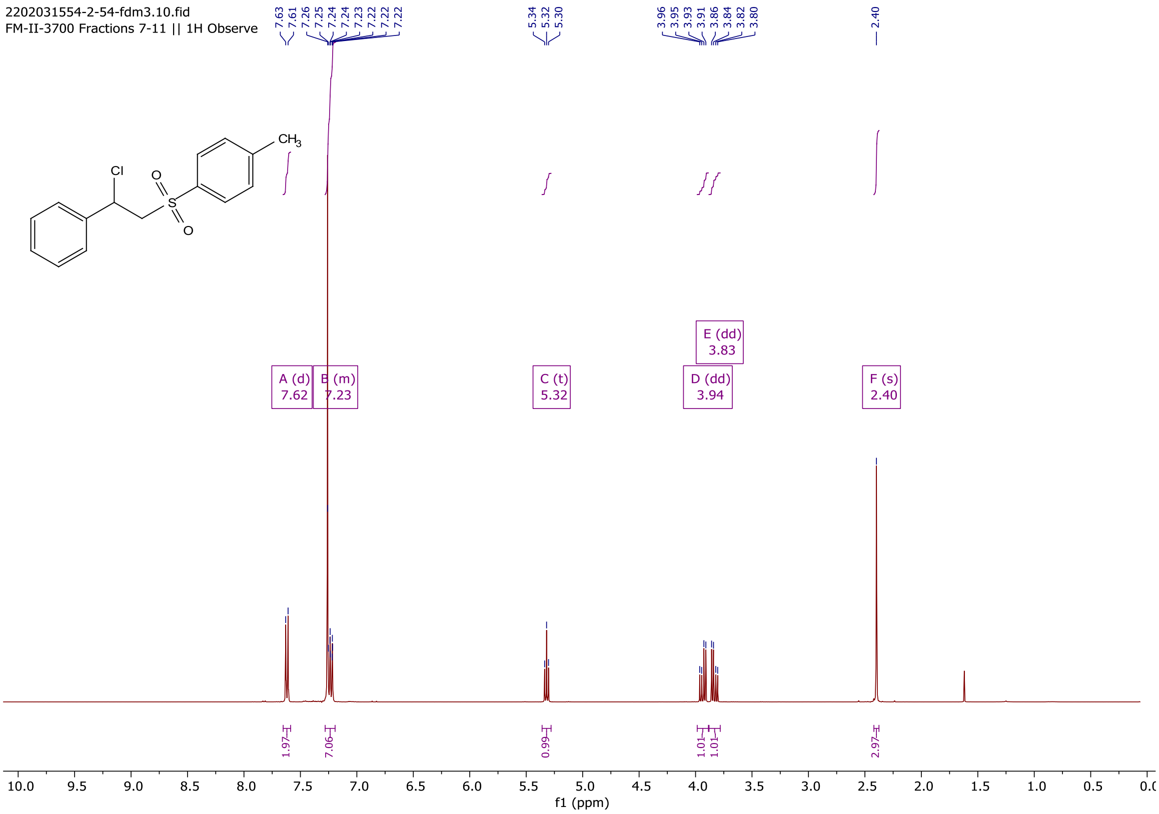

Figure **S30**. ^{1}H NMR spectrum of 1-((2-chloro-2-phenylethyl)sulfonyl)-4-methylbenzene in $CDCl_3$ at 400 MHz.

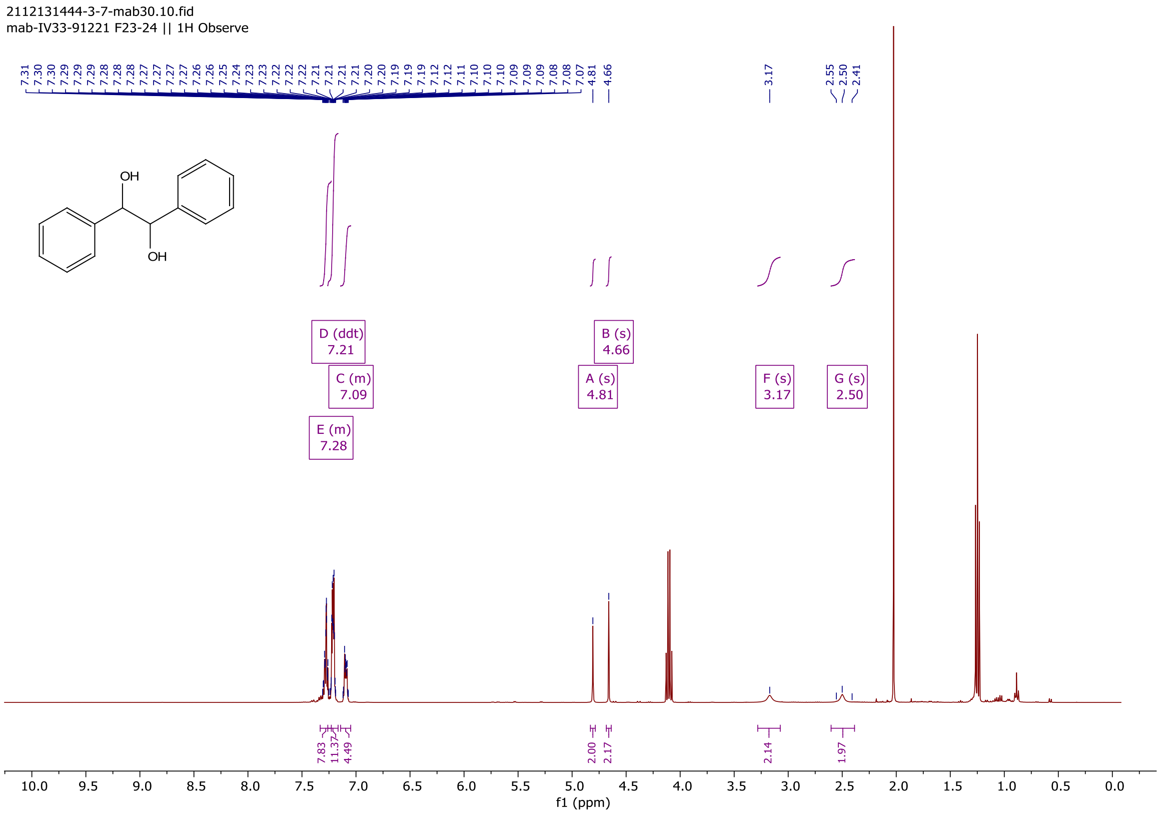

Figure **S31**. ^{1}H NMR spectrum of 1,2-diphenylethane-1,2-diol in $CDCl_3$ at 400 MHz.

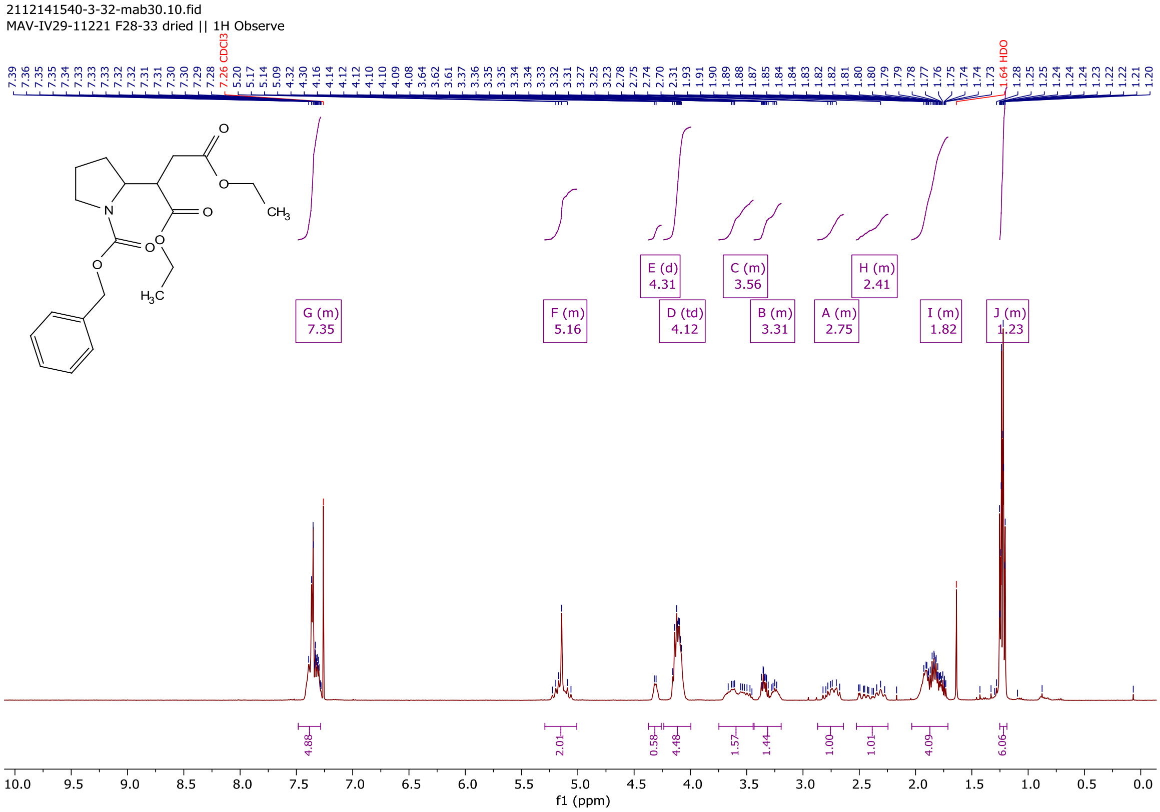

Figure **S32**. ^{1}H NMR spectrum of diethyl 2-(1-((benzyloxy)carbonyl)34yrrolidine-2-yl)succinate in $CDCl_3$ at 400 MHz.

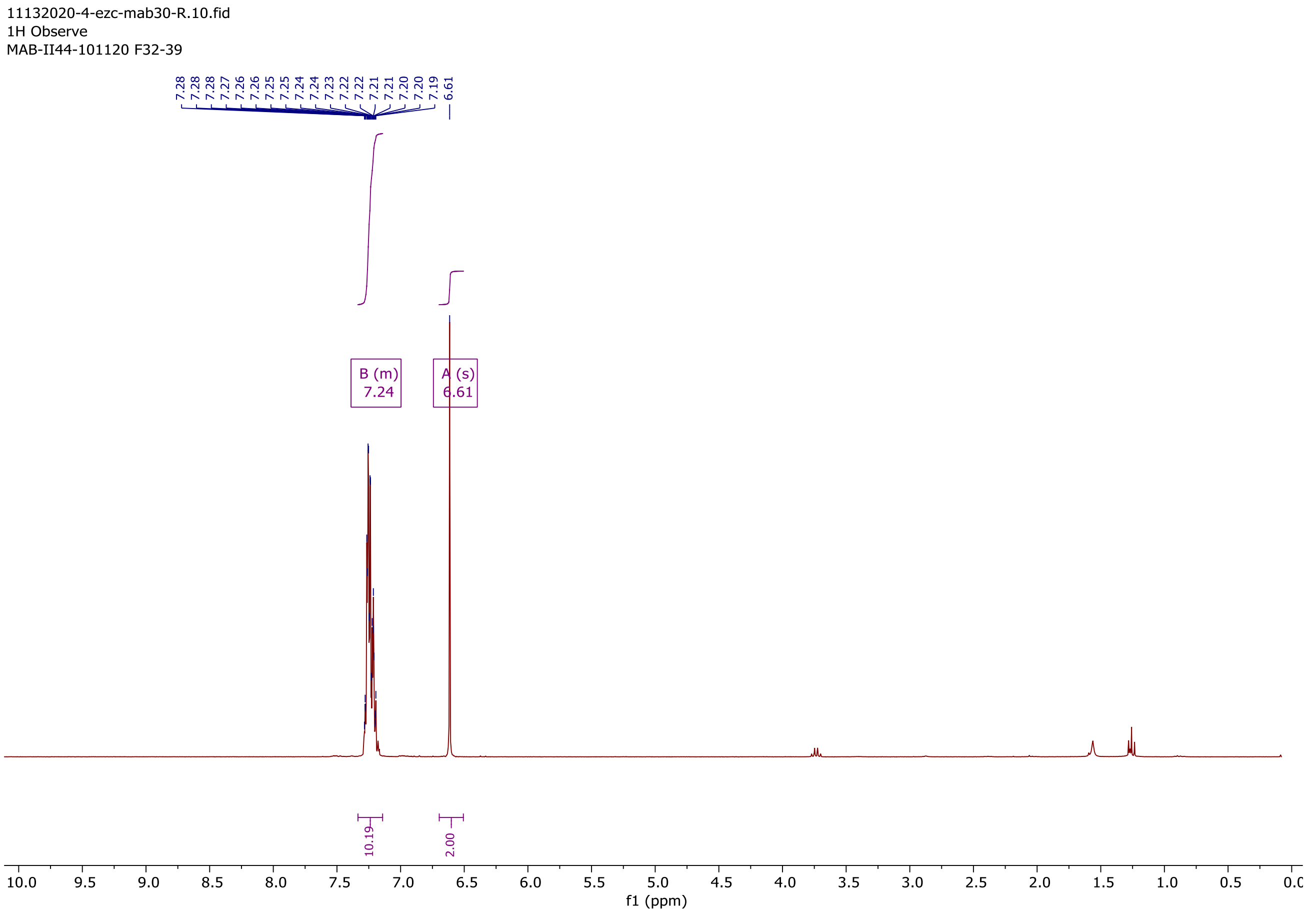

Figure **S33**. ^{1}H NMR spectrum of *Z*-stilbene in $CDCl_3$ at 400 MHz.

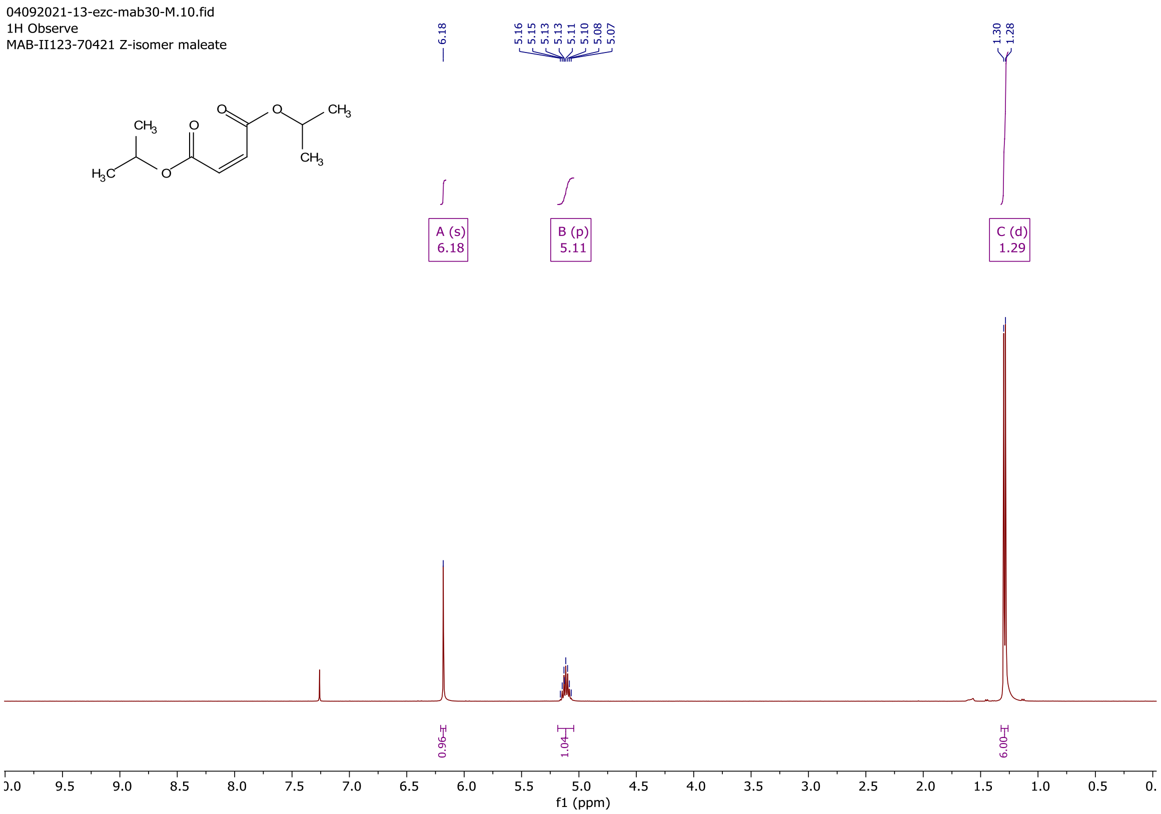

Figure **S34**. ^{1}H NMR spectrum of di*iso*propyl maleate in $CDCl_3$ at 400 MHz.

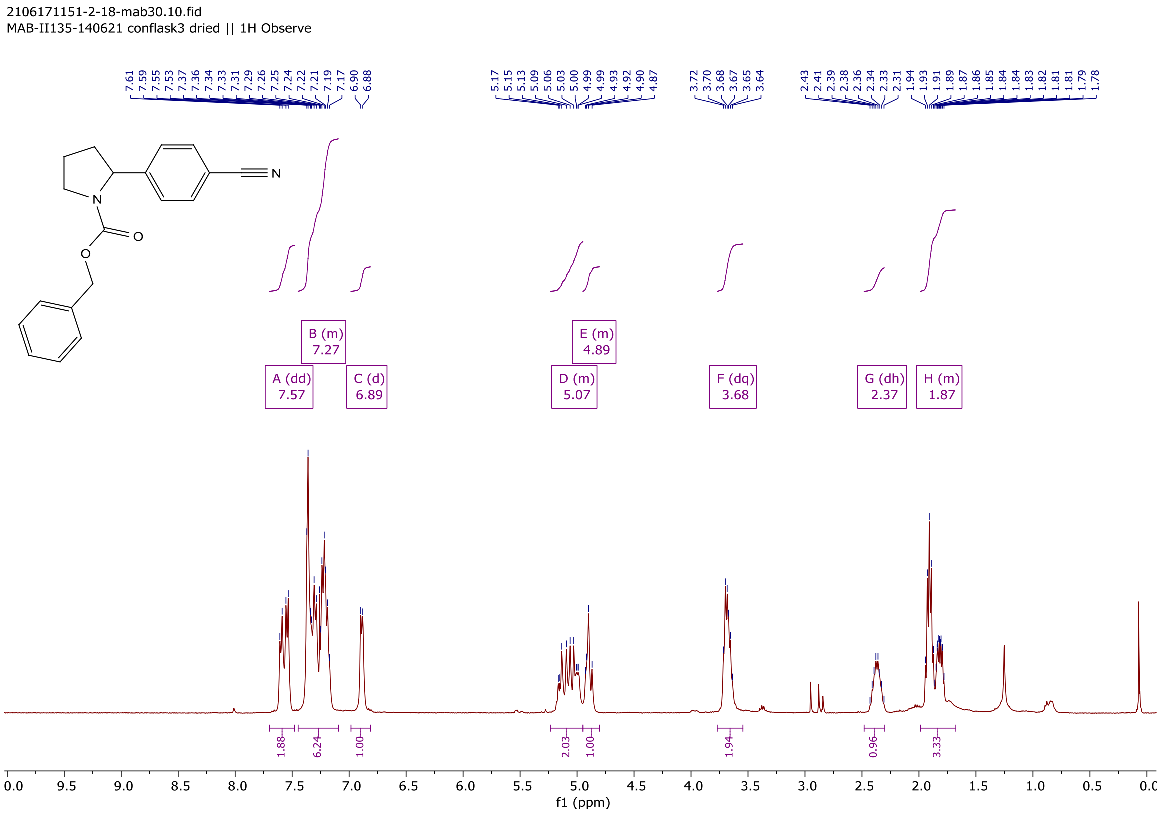

Figure **S35**. ^{1}H NMR spectrum of benzyl 2-(4-cyanophenyl)pyrrolidine-1-carboxylate in $CDCl_3$ at 400 MHz.

DFT calculations

Table **S8**. Selected data from DFT calculations for **pDTCz-DPmS**.

Selected data from DFT calculations:	MeCN	DCM	THF	DMF
HOMO / eV	-6.08	-6.06	-6.05	-6.08
LUMO / eV	-1.90	-1.89	-1.89	-1.90
$\Delta E_{HOMO-LUMO}$ / eV	4.18	4.17	4.16	4.18
Ground state dipole moment magnitude / D	5.91	5.75	5.72	5.91
S_1 / eV	3.47	3.47	3.47	3.47
T_1 / eV	2.98	2.97	2.97	2.98
ΔE_{ST} / eV	0.49	0.50	0.50	0.49
Dipole moment in the T_1 state / D	16.32	19.56	15.22	16.32
Predicted Phosphorescence at RT / eV	2.71	2.51	2.75	2.71
Imaginary frequencies	0	0	0	0

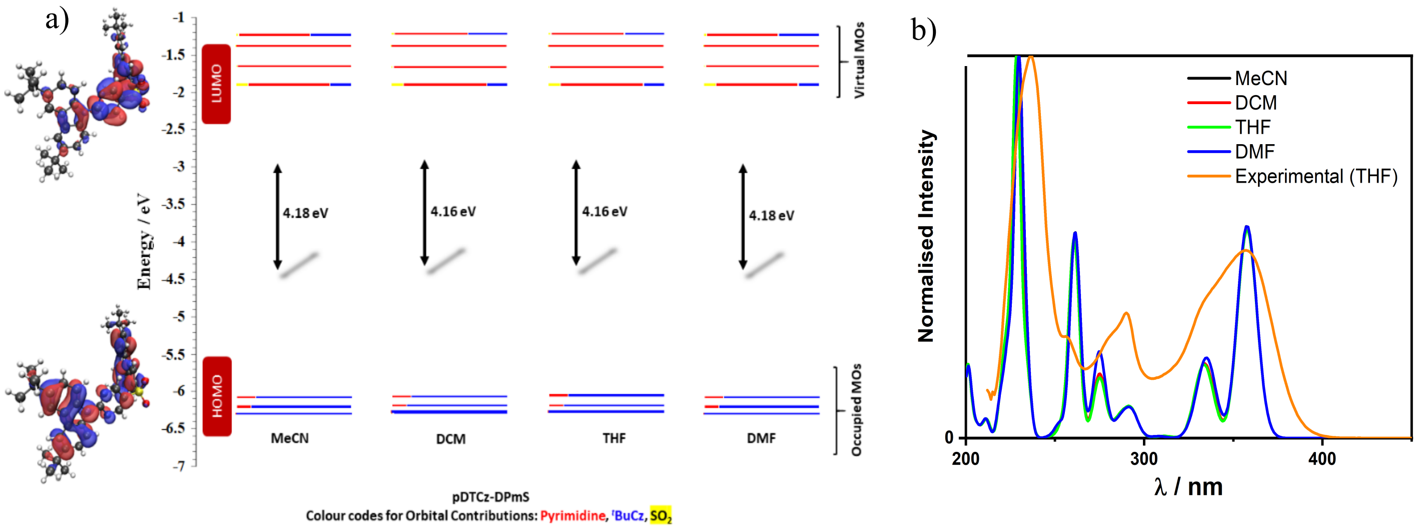

Figure **S36**. (a) Orbital contributions calculated from DFT results in each of the solvents modelled (MeCN, DCM, THF and DMF) for **pDTCz-DPmS** and (b) the simulated UV-Vis absorption spectra of **pDTCz-DPmS** from DFT calculations in MeCN, DCM, THF and DMF compared with the experimental data obtained in THF. Simulated spectra were generated with a full-width at half maximum set to 1000 cm^{-1}.

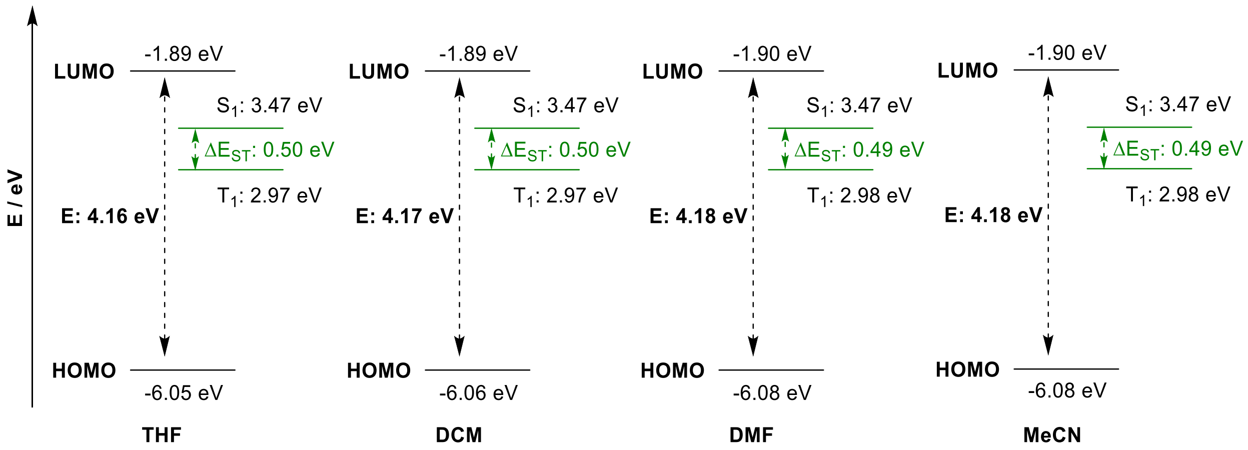

Figure **S37**. Energy level diagram for **pDTCz-DPmS** in the different solvents.

Cartesian coordinates of **pDTCz-DPmS** in THF

Element	X Coord	Y Coord	Z Coord
S	0.0000010	0.0001030	4.2336010
O	0.0224710	1.2772260	4.9491880
N	2.9794050	-1.2251740	1.8642430
N	3.0945490	1.1577870	1.9890660
N	4.5966140	-0.0428330	0.6951070
C	1.9299920	-1.2094640	2.6676050
H	1.4976600	-2.1699460	2.9399890
C	1.3996950	-0.0192120	3.1629480
C	2.0437800	1.1588680	2.7911890
H	1.7026630	2.1209580	3.1666050
C	3.5177370	-0.0364830	1.5442090
C	5.3004380	1.1003120	0.2334440
C	5.1304740	2.4580900	0.4846200
H	4.3482150	2.8115870	1.1385480
C	6.0022940	3.3542480	-0.1322570
H	5.8574160	4.4086890	0.0735550
C	7.0364680	2.9518880	-0.9887250
C	7.1874670	1.5832300	-1.2251510
H	7.9740860	1.2195440	-1.8802780
C	6.3318380	0.6691510	-0.6232720
C	6.2667690	-0.7731640	-0.6937490
C	7.0452660	-1.6956950	-1.3898430
H	7.8599410	-1.3353480	-2.0081800
C	6.7736510	-3.0575220	-1.2862010
C	5.7009790	-3.4491930	-0.4650270
H	5.4679430	-4.5053920	-0.3655030
C	4.9089620	-2.5500970	0.2388170
H	4.0952450	-2.8945730	0.8583750
C	5.1999430	-1.1913570	0.1192840
C	7.9868130	3.9438980	-1.6595460
C	7.6720690	5.3933190	-1.2855900
H	6.6624380	5.6825210	-1.5950610

H	7.7652730	5.5640990	-0.2081590
H	8.3775210	6.0611990	-1.7896230
C	7.8716410	3.8030160	-3.1849140
H	6.8524330	4.0205590	-3.5200260
H	8.5522370	4.5030050	-3.6817580
H	8.1277370	2.7931910	-3.5189090

Cartesian coordinates of **pDTCz-DPmS** in DCM

Element	X Coord	Y Coord	Z Coord
S	0.0000000	0.0000330	4.2290140
O	0.0225340	1.2768860	4.9453340
N	2.9808350	-1.2254980	1.8618250
N	3.0950860	1.1575310	1.9854290
N	4.5986710	-0.0432820	0.6934380
C	1.9308280	-1.2098190	2.6644720
H	1.4985250	-2.1703200	2.9367240
C	1.4000430	-0.0194130	3.1589590
C	2.0437580	1.1587700	2.7868670
H	1.7019140	2.1210260	3.1611260
C	3.5189590	-0.0368240	1.5416640
C	5.3036860	1.0997030	0.2332940
C	5.1340050	2.4575110	0.4847430
H	4.3508060	2.8112280	1.1374430
C	6.0072920	3.3535130	-0.1303600
H	5.8625420	4.4079340	0.0756490
C	7.0426720	2.9509920	-0.9853590
C	7.1933570	1.5823310	-1.2221870
H	7.9807910	1.2185000	-1.8762510
C	6.3362610	0.6684300	-0.6220230
C	6.2705070	-0.7738280	-0.6933290
C	7.0494730	-1.6964130	-1.3889180
H	7.8653030	-1.3361780	-2.0057990
C	6.7766970	-3.0581450	-1.2866710
C	5.7023930	-3.4496520	-0.4674780

H	5.4682870	-4.5057320	-0.3692000
C	4.9099670	-2.5504780	0.2358690
H	4.0950310	-2.8949160	0.8538650
C	5.2021750	-1.1918180	0.1179000
C	7.9945760	3.9428450	-1.6542180
C	7.6800740	5.3922320	-1.2799260
H	6.6710900	5.6822040	-1.5907900
H	7.7716830	5.5623670	-0.2022530
H	8.3867340	6.0599190	-1.7825150
C	7.8816250	3.8028160	-3.1798380
H	6.8630910	4.0213600	-3.5163950
H	8.5634730	4.5026250	-3.6751990
H	8.1375750	2.7929940	-3.5139600

C	9.4351790	3.6336900	-1.2194350
H	10.1318830	4.3324890	-1.6951740
H	9.5430280	3.7284950	-0.1341840
H	9.7343250	2.6196070	-1.5005960
C	7.5933190	-4.1204170	-2.0211310
C	8.2257670	-5.0729360	-0.9952130
H	8.8109830	-5.8447700	-1.5069070
H	8.8935100	-4.5288940	-0.3195460
H	7.4675030	-5.5755180	-0.3876790
C	6.6690830	-4.9162550	-2.9550650
H	7.2404170	-5.6855270	-3.4859150
H	5.8676300	-5.4161270	-2.4032420
H	6.2077280	-4.2585620	-3.6987130
C	8.7135600	-3.5059310	-2.8619480
H	9.2691230	-4.3015230	-3.3678930
H	8.3206690	-2.8338250	-3.6319170
H	9.4236030	-2.9461390	-2.2443980
O	-0.0225340	-1.2768040	4.9453620
N	-2.9808360	1.2255120	1.8618010
N	-3.0950860	-1.1575140	1.9854550
N	-4.5986710	0.0432760	0.6934380
C	-1.9308290	1.2098500	2.6644470
H	-1.4985260	2.1703570	2.9366790
C	-1.4000430	0.0194550	3.1589590
C	-2.0437570	-1.1587360	2.7868920
H	-1.7019120	-2.1209840	3.1611700
C	-3.5189600	0.0368310	1.5416650
C	-5.3036890	-1.0997120	0.2333020
C	-5.1340100	-2.4575190	0.4847580
H	-4.3508130	-2.8112350	1.1374600
C	-6.0072980	-3.3535230	-0.1303400
H	-5.8625500	-4.4079440	0.0756740
C	-7.0426780	-2.9510050	-0.9853420
C	-7.1933590	-1.5823450	-1.2221780
H	-7.9807930	-1.2185160	-1.8762440
C	-6.3362620	-0.6684430	-0.6220180
C	-6.2705060	0.7738140	-0.6933340
C	-7.0494700	1.6963980	-1.3889280
H	-7.8653010	1.3361600	-2.0058080
C	-6.7766910	3.0581300	-1.2866900

C	-5.7023870	3.4496390	-0.4674990
H	-5.4682790	4.5057190	-0.3692270
C	-4.9099620	2.5504680	0.2358540
H	-4.0950250	2.8949070	0.8538480
C	-5.2021740	1.1918080	0.1178920
C	-7.9945840	-3.9428600	-1.6541960
C	-7.6800830	-5.3922460	-1.2798970
H	-6.6711000	-5.6822200	-1.5907610
H	-7.7716910	-5.5623760	-0.2022230
H	-8.3867440	-6.0599340	-1.7824820
C	-7.8816340	-3.8028390	-3.1798160
H	-6.8631010	-4.0213850	-3.5163730
H	-8.5634840	-4.5026500	-3.6751730
H	-8.1375840	-2.7930180	-3.5139430
C	-9.4351850	-3.6337010	-1.2194130
H	-10.1318910	-4.3325010	-1.6951480
H	-9.5430340	-3.7285010	-0.1341600
H	-9.7343300	-2.6196190	-1.5005780
C	-7.5933110	4.1203990	-2.0211550
C	-8.2257390	5.0729370	-0.9952430
H	-8.8109530	5.8447700	-1.5069410
H	-8.8934810	4.5289100	-0.3195620
H	-7.4674640	5.5755190	-0.3877230
C	-6.6690790	4.9162160	-2.9551110
H	-7.2404130	5.6854840	-3.4859670
H	-5.8676170	5.4160920	-2.4033030
H	-6.2077360	4.2585080	-3.6987530
C	-8.7135670	3.5059100	-2.8619510
H	-9.2691200	4.3015000	-3.3679120
H	-8.3206910	2.8337800	-3.6319070

Cartesian coordinates of **pDTCz-DPmS** in DMF

Element	X Coord	Y Coord	Z Coord
S	0.0000020	0.0001400	4.2037210
O	0.0218390	1.2759860	4.9229360
N	2.9932990	-1.2270170	1.8545920
N	3.0958230	1.1571840	1.9614040

N	4.6111740	-0.0460740	0.6853550
C	1.9395690	-1.2109450	2.6526170
H	1.5105180	-2.1715200	2.9289910
C	1.4021410	-0.0193290	3.1368670
C	2.0412550	1.1595590	2.7587750
H	1.6930690	2.1231590	3.1233690
C	3.5268470	-0.0383140	1.5285150
C	5.3236900	1.0953980	0.2336390
C	5.1578930	2.4532650	0.4883580
H	4.3702800	2.8085860	1.1349520
C	6.0403480	3.3476080	-0.1162960
H	5.8982270	4.4019280	0.0920970
C	7.0811900	2.9434420	-0.9641750
C	7.2278070	1.5748550	-1.2048300
H	8.0189260	1.2096960	-1.8536860
C	6.3616050	0.6626690	-0.6147620
C	6.2902430	-0.7791080	-0.6915490
C	7.0697240	-1.7025460	-1.3858400
H	7.8917370	-1.3436290	-1.9952560
C	6.7887310	-3.0633770	-1.2922540
C	5.7057770	-3.4530250	-0.4833010
H	5.4644520	-4.5081430	-0.3925770
C	4.9131570	-2.5529180	0.2188930
H	4.0918370	-2.8963820	0.8290200
C	5.2139250	-1.1951340	0.1103940
C	8.0430220	3.9334920	-1.6214550
C	7.7326170	5.3827900	-1.2433950
H	6.7276010	5.6791650	-1.5610040
H	7.8163090	5.5479050	-0.1642850
H	8.4467390	6.0488130	-1.7375360
C	7.9412750	3.8001830	-3.1484970
H	6.9266530	4.0261340	-3.4921060
H	8.6307910	4.4984000	-3.6353410
H	8.1945690	2.7903710	-3.4846800

C	9.4787520	3.6153600	-1.1770070
H	10.1820630	4.3130010	-1.6445640
H	9.5788610	3.7055640	-0.0905490
H	9.7752500	2.6011230	-1.4604460
C	7.6044820	-4.1264760	-2.0265420
C	8.2207260	-5.0908260	-1.0017500
H	8.8050840	-5.8628130	-1.5141100
H	8.8865870	-4.5564720	-0.3164430
H	7.4535600	-5.5928040	-0.4049320
C	6.6827100	-4.9092550	-2.9739430
H	7.2532450	-5.6794680	-3.5042260
H	5.8725070	-5.4063910	-2.4324820
H	6.2336830	-4.2433770	-3.7179410
C	8.7366340	-3.5143230	-2.8529690
H	9.2912440	-4.3107310	-3.3586450
H	8.3556910	-2.8345990	-3.6222350
H	9.4446360	-2.9631690	-2.2253810
O	-0.0218360	-1.2756640	4.9230110
N	-2.9933100	1.2271550	1.8545360
N	-3.0958070	-1.1570420	1.9614590
N	-4.6111720	0.0461350	0.6853550
C	-1.9395790	1.2111320	2.6525620
H	-1.5105380	2.1717240	2.9288900
C	-1.4021380	0.0195440	3.1368670
C	-2.0412400	-1.1593670	2.7588300
H	-1.6930430	-2.1229470	3.1234690
C	-3.5268440	0.0384320	1.5285140
C	-5.3236530	-1.0953750	0.2336800
C	-5.1578110	-2.4532270	0.4884470
H	-4.3701860	-2.8084990	1.1350540
C	-6.0402360	-3.3476210	-0.1161750
H	-5.8980790	-4.4019290	0.0922540
C	-7.0810920	-2.9435190	-0.9640680
C	-7.2277540	-1.5749450	-1.2047710
H	-8.0188860	-1.2098340	-1.8536390
C	-6.3615820	-0.6627090	-0.6147350
C	-6.2902660	0.7790670	-0.6915730
C	-7.0697780	1.7024550	-1.3858960
H	-7.8917790	1.3434890	-1.9952990
C	-6.7888300	3.0632980	-1.2923590

C	-5.7058890	3.4530100	-0.4834190
H	-5.4645990	4.5081400	-0.3927310
C	-4.9132380	2.5529540	0.2188050
H	-4.0919290	2.8964680	0.8289190
C	-5.2139600	1.1951560	0.1103530
C	-8.0428920	-3.9336230	-1.6213120
C	-7.7324390	-5.3828980	-1.2432010
H	-6.7274130	-5.6792510	-1.5608000
H	-7.8161240	-5.5479780	-0.1640850
H	-8.4465380	-6.0489620	-1.7373180
C	-7.9411510	-3.8003650	-3.1483590
H	-6.9265220	-4.0262940	-3.4919610
H	-8.6306440	-4.4986230	-3.6351780
H	-8.1944800	-2.7905740	-3.4845770
C	-9.4786310	-3.6155240	-1.1768740
H	-10.1819200	-4.3132040	-1.6444050
H	-9.5787360	-3.7056930	-0.0904130
H	-9.7751640	-2.6013070	-1.4603480
C	-7.6046170	4.1263440	-2.0266840
C	-8.2208840	5.0907150	-1.0019270
H	-8.8052680	5.8626640	-1.5143140
H	-8.8867270	4.5563690	-0.3165970
H	-7.4537310	5.5927400	-0.4051320
C	-6.6828720	4.9091130	-2.9741200
H	-7.2534330	5.6792870	-3.5044310
H	-5.8726830	5.4062970	-2.4326830
H	-6.2338280	4.2432200	-3.7180930
C	-8.7367540	3.5141240	-2.8530820
H	-9.2913870	4.3104960	-3.3587910
H	-8.3557940	2.8343780	-3.6223200
H	-9.4447380	2.9629770	-2.2254690

Cartesian coordinates of **pDTCz-DPmS** in MeCN

Element	X Coord	Y Coord	Z Coord
S	0.0000020	0.0001410	4.2040940
O	0.0218770	1.2760010	4.9232680
N	2.9930120	-1.2269980	1.8545560

N	3.0958960	1.1571670	1.9618780
N	4.6109840	-0.0460300	0.6854660
C	1.9393410	-1.2109370	2.6526560
H	1.5101630	-2.1715100	2.9288540
C	1.4021060	-0.0193470	3.1371880
C	2.0413760	1.1595200	2.7593070
H	1.6933490	2.1230890	3.1241410
C	3.5267250	-0.0383010	1.5287010
C	5.3233740	1.0954760	0.2336250
C	5.1574870	2.4533420	0.4882750
H	4.3699370	2.8086290	1.1349620
C	6.0397830	3.3477230	-0.1165490
H	5.8975990	4.4020450	0.0917940
C	7.0805550	2.9435960	-0.9645280
C	7.2272630	1.5750080	-1.2051080
H	8.0183350	1.2098780	-1.8540390
C	6.3612170	0.6627830	-0.6148770
C	6.2899650	-0.7790030	-0.6915730
C	7.0694700	-1.7024190	-1.3858600
H	7.8913920	-1.3434710	-1.9953810
C	6.7886320	-3.0632700	-1.2921280
C	5.7058090	-3.4529620	-0.4830260
H	5.4646190	-4.5081000	-0.3921740
C	4.9131600	-2.5528780	0.2191610
H	4.0919350	-2.8963680	0.8294000
C	5.2137660	-1.1950740	0.1105000
C	8.0422140	3.9336870	-1.6219970
C	7.7317180	5.3829840	-1.2440070
H	6.7266330	5.6792370	-1.5615120
H	7.8155310	5.5481880	-0.1649190
H	8.4457090	6.0490430	-1.7382870
C	7.9402930	3.8002600	-3.1490160
H	6.9256050	4.0260730	-3.4925170
H	8.6296770	4.4985130	-3.6359990
H	8.1936490	2.7904490	-3.4851580

C	9.4780270	3.6157280	-1.1776970
H	10.1812200	4.3133970	-1.6453890
H	9.5782560	3.7060150	-0.0912580
H	9.7745860	2.6014970	-1.4610920
C	7.6044350	-4.1263470	-2.0263890
C	8.2209210	-5.0904950	-1.0015550
H	8.8053150	-5.8624760	-1.5138830
H	8.8867880	-4.5559740	-0.3163870
H	7.4538840	-5.5924880	-0.4045840
C	6.6826620	-4.9093510	-2.9736010
H	7.2532370	-5.6795430	-3.5038730
H	5.8725890	-5.4065430	-2.4319960
H	6.2334500	-4.2436120	-3.7176110
C	8.7364150	-3.5141410	-2.8530140
H	9.2910670	-4.3105310	-3.3586730
H	8.3552940	-2.8345470	-3.6223070
H	9.4444210	-2.9628360	-2.2255660
O	-0.0218730	-1.2756770	4.9233410
N	-2.9930220	1.2271420	1.8545010
N	-3.0958800	-1.1570190	1.9619310
N	-4.6109810	0.0460960	0.6854660
C	-1.9393500	1.2111280	2.6526010
H	-1.5101820	2.1717180	2.9287560
C	-1.4021030	0.0195660	3.1371880
C	-2.0413610	-1.1593250	2.7593610
H	-1.6933230	-2.1228730	3.1242380
C	-3.5267220	0.0384240	1.5287000
C	-5.3233340	-1.0954500	0.2336670
C	-5.1573980	-2.4533000	0.4883660
H	-4.3698350	-2.8085360	1.1350660
C	-6.0396620	-3.3477340	-0.1164260
H	-5.8974400	-4.4020440	0.0919540
C	-7.0804490	-2.9436740	-0.9644190
C	-7.2272070	-1.5751000	-1.2050470
H	-8.0182910	-1.2100210	-1.8539910
C	-6.3611920	-0.6628230	-0.6148490
C	-6.2899890	0.7789620	-0.6915980
C	-7.0695280	1.7023260	-1.3859180
H	-7.8914380	1.3433260	-1.9954240
C	-6.7887380	3.0631900	-1.2922340

C	-5.7059290	3.4529490	-0.4831470
H	-5.4647770	4.5080990	-0.3923320
C	-4.9132460	2.5529190	0.2190710
H	-4.0920330	2.8964600	0.8292960
C	-5.2138030	1.1950990	0.1104590
C	-8.0420740	-3.9338230	-1.6218500
C	-7.7315250	-5.3830960	-1.2438080
H	-6.7264300	-5.6793240	-1.5613040
H	-7.8153320	-5.5482630	-0.1647150
H	-8.4454940	-6.0491980	-1.7380630
C	-7.9401600	-3.8004480	-3.1488750
H	-6.9254650	-4.0262360	-3.4923680
H	-8.6295190	-4.4987430	-3.6358310
H	-8.1935540	-2.7906580	-3.4850520
C	-9.4778980	-3.6158990	-1.1775600
H	-10.1810670	-4.3136110	-1.6452250
H	-9.5781220	-3.7061500	-0.0911180
H	-9.7744930	-2.6016890	-1.4609910
C	-7.6045800	4.1262110	-2.0265340
C	-8.2210930	5.0903790	-1.0017350
H	-8.8055160	5.8623210	-1.5140920
H	-8.8869400	4.5558630	-0.3165430
H	-7.4540710	5.5924220	-0.4047870
C	-6.6828370	4.9092090	-2.9737810
H	-7.2534400	5.6793590	-3.5040820
H	-5.8727790	5.4064490	-2.4321990
H	-6.2336050	4.2434550	-3.7177660
C	-8.7365430	3.5139350	-2.8531300
H	-9.2912210	4.3102870	-3.3588210
H	-8.3554020	2.8343210	-3.6223960
H	-9.4445300	2.9626340	-2.2256570

References

(1) Etherington, M. K.; Kukhta, N. A.; Higginbotham, H. F.; Danos, A.; Bismillah, A. N.; Graves, D. R.; McGonigal, P. R.; Haase, N.; Morherr, A.; Batsanov, A. S.; Pflumm, C.; Bhalla, V.; Bryce, M. R.; Monkman, A. P. Persistent Dimer Emission in Thermally Activated Delayed Fluorescence Materials. *J. Phys. Chem. C* **2019**, *123* (17), 11109–11117. https://doi.org/10.1021/acs.jpcc.9b01458.

(2) Wong, M. Y.; Krotkus, S.; Copley, G.; Li, W.; Murawski, C.; Hall, D.; Hedley, G. J.; Jaricot, M.; Cordes, D. B.; Slawin, A. M. Z.; Olivier, Y.; Beljonne, D.; Muccioli, L.; Moral, M.; Gather, M. C.; Samuel, I. D. W.; Zysman-colman, E. Deep-Blue Oxadiazole-Containing Thermally Activated Delayed Fluorescence Emitters for Organic Light-Emitting Diodes. *ACS Appl. Mater. Interfaces* **2018**, *10*, 33360–33372. https://doi.org/10.1021/acsami.8b11136.

(3) Hu, C.; Chen, Y. Chemoselective and Fast Decarboxylative Allylation by Photoredox Catalysis under Mild Conditions. *Org. Chem. Front.* **2015**, *2*, 1352–1355. https://doi.org/10.1039/c5qo00187k.

(4) Vasu, D.; Fuentes de Arriba, A. L.; Leitch, J. A.; De Gombert, A.; Dixon, D. J. Primary α-Tertiary Amine Synthesis via α-C-H Functionalization. *Chem. Sci.* **2019**, *10* (11), 3401–3407. https://doi.org/10.1039/c8sc05164j.

(5) Rawner, T.; Lutsker, E.; Kaiser, C. A.; Reiser, O. The Different Faces of Photoredox Catalysts: Visible-Light-Mediated Atom Transfer Radical Addition (ATRA) Reactions of Perfluoroalkyl Iodides with Styrenes and Phenylacetylenes. *ACS Catal.* **2018**, *8* (5), 3950–3956. https://doi.org/10.1021/acscatal.8b00847.

(6) Wang, C.; Guo, M.; Qi, R.; Shang, Q.; Liu, Q.; Wang, S.; Zhao, L.; Wang, R.; Xu, Z. Visible-Light-Driven, Copper-Catalyzed Decarboxylative C(Sp3)−H Alkylation of Glycine and Peptides. *Angew. Chemie Int. Ed.* **2018**, *57* (48), 15841–15846. https://doi.org/10.1002/anie.201809400.

(7) Peterson, A.; Kaasik, M.; Metsala, A.; Järving, I.; Adamson, J.; Kanger, T. Tunable Chiral Triazole-Based Halogen Bond Donors: Assessment of Donor Strength in Solution with Nitrogen-Containing Acceptors. *RSC Adv.* **2019**, *9* (21), 11718–11721. https://doi.org/10.1039/c9ra01692a.

(8) Cornella, J.; Edwards, J. T.; Qin, T.; Kawamura, S.; Wang, J.; Pan, C. M.; Gianatassio, R.; Schmidt, M.; Eastgate, M. D.; Baran, P. S. Practical Ni-Catalyzed Aryl-Alkyl Cross-Coupling of Secondary Redox-Active Esters. *J. Am. Chem. Soc.* **2016**, *138* (7), 2174–2177. https://doi.org/10.1021/jacs.6b00250.

(9) Schneider, L. M.; Schmiedel, V. M.; Pecchioli, T.; Lentz, D.; Merten, C.; Christmann, M. Asymmetric Synthesis of Carbocyclic Propellanes. *Org. Lett.* **2017**, *19* (9), 2310–2313. https://doi.org/10.1021/acs.orglett.7b00836.

(10) Katakam, N. K.; Seifert, C. W.; D'Auria, J.; Li, G. Efficient Synthesis of Methyl (S)-4-(1-Methylpyrrolidin-2-YL)-3-Oxobutanoate as the Key Intermediate for Tropane Alkaloid Biosynthesis with Optically Acitve Form. *Heterocycles* **2019**, *99* (1), 604–613. https://doi.org/10.3987/COM-18-S(F)4.

(11) Demas, J. N.; Crosby, G. A. The Measurement of Photoluminescence Quantum Yields. A Review. *J. Phys* **1971**, *75*, 991–1024.

(12) Suzuki, K.; Kobayashi, A.; Kaneko, S.; Takehira, K.; Yoshihara, T.; Ishida, H.; Shiina, Y.; Oishi, S.; Tobita, S. Reevaluation of Absolute Luminescence Quantum Yields of Standard Solutions Using a Spectrometer with an Integrating Sphere and a Back-Thinned CCD Detector. *Phys. Chem. Chem. Phys.* **2009**, *11* (42), 9850–9860. https://doi.org/10.1039/b912178a.

(13) Pavlishchuk, V. V.; Addison, A. W. Conversion Constants for Redox Potentials Measured versus Different Reference Electrodes in Acetonitrile Solutions at 25°C. *Inorganica Chim. Acta* **2000**, *298* (1), 97–102. https://doi.org/10.1016/S0020-1693(99)00407-7.

(14) Connelly, N. G.; Geiger, W. E. Chemical Redox Agents for Organometallic Chemistry. *Chem. Rev.* **1996**, *96* (2), 877–910. https://doi.org/10.1021/cr940053x.

(15) Frisch, M. J.; W, T. G.; Schlegel, H. B.; Scuseria, G. E.; Robb, M. A.; Cheeseman, J. R.; Scalmani, G.; Barone, V.; Peterssom, G. A.; Nakatsuji, H.; Li, X.; Caricato, M.; Marenich, A. V; Bloino, J.; Janesko, B. G.; Gomperts, R.; Mennucci, B.; Hratchian, H. P.; Ortiz, J. V; Izmaylov, A. F.; Sonnenberg, J. L.; Williams-Young, D.; Ding, F.; Lipparini, F.; Egidi, F.; Goings, J.; Peng, B.; Petrone, A.; Henderson, T.; Ranasinghe, D.; Zakrzewski, V. G.; Gao, J.; Rega, N.; Zheng, G.; Liang, W.; Hada, M.; Ehara, M.; Toyota, K.; Fukuda, R.; Hasegawa, J.; Ishida, M.; Nakajima, T.;

Honda, Y.; Kitao, O.; Nakai, H.; Vreven, T.; Throssell, K.; Montgomery Jr, J. A.; Peralta, J. E.; Ogliaro, F.; Bearpark, M. J.; Heyd, J. J.; Brothers, E. N.; Kudin, K. N.; Staroverov, V. N.; Keith, T. A.; Kobayashi, R.; Normand, J.; Raghavachari, K.; Rendell, A. P.; Burant, J. C.; Iyengar, S. S.; Tomasi, J.; Cossi, M.; Millam, J. M.; Klene, M.; Adamo, C.; Cammi, R.; Ochterski, J. W.; Martin, R. L.; Morokuma, K.; Farkas, O.; Foresman, J. B.; Fox, D. J. Gaussian 16, Revision C.01. Gaussian Inc: Wallingford CT 2019.

(16) Adamo, C.; Barone, V. Toward Reliable Density Functional Methods without Adjustable Parameters: The PBE0 Model. *J. Chem. Phys.* **1999**, *110* (13), 6158–6170. https://doi.org/10.1063/1.478522.

(17) Dunning, T. H. Gaussian Basis Sets for Use in Correlated Molecular Calculations. I. The Atoms Boron through Neon and Hydrogen. *J. Chem. Phys.* **1989**, *90* (2), 1007–1023. https://doi.org/10.1063/1.456153.

(18) O'Boyle, N. M.; Tenderholt, A. L.; Langner, K. M. Cclib: A Library for Package-Independent Computational Chemistry Algorithms. *J. Comput. Chem.* **2008**, *29* (5), 839–845. https://doi.org/10.1002/jcc.20823.

(19) Humphrey, W.; Dalke, A.; Schulten, K. VMD: Visual Molecular Dynamics. *J. Mol. Graph.* **1996**, *14*, 33–38.

(20) Edward, J.; Ercal, F.; Walters, F. G.; Pottinger, H. J. AN EFFICIENT LIBRARY FOR PARALLEL RAY TRACING AND ANIMATION, University of Missouri-Rolla, 1998.

(21) Hunter, J. D. Matplotlib: A 2D Graphics Environment. *Comput. Sci. Eng.* **2007**, *9*, 90–95.

(22) Mansencal, T.; Mauderer, M.; Parsons, M.; Shaw, N.; Wheatley, K.; Cooper, S.; Vandenberg, J. D.; Canavan, L.; Crowson, K.; Lev, O.; Leinweber, K.; Sharma, S.; Sobotka, T. J.; Moritz, D.; Pppp, M.; Rane, C.; Eswaramoorthy, P.; Mertic, J.; Pearlstine, B.; Leonhardt, M.; Niemitalo, O.; Szymanski, M.; Schambach, M.; Huang, S.; Wei, M.; Joywardhan, N.; Wagih, O.; Redman, P.; Goldstone, J.; Hill, S. Colour 0.3.16. **2020**. https://doi.org/10.5281/ZENODO.3757045.

(23) Bayer, M. https://www.makotemplates.org, (accessed May 2020).

(24) Community, K. https://weasyprint.org, (accessed May 2020).

(25) Virtanen, P.; Gommers, R.; Oliphant, T. E.; Haberland, M.; Reddy, T.; Cournapeau, D.; Burovski, E.; Peterson, P.; Weckesser, W.; Bright, J.; van der Walt, S. J.; Brett, M.; Wilson, J.; Millman, K. J.; Mayorov, N.; Nelson, A. R. J.; Jones, E.; Kern, R.; Larson, E.; Carey, C. J.; Polat, İ.; Feng, Y.; Moore, E. W.; VanderPlas, J.; Laxalde, D.; Perktold, J.; Cimrman, R.; Henriksen, I.; Quintero, E. A.; Harris, C. R.; Archibald, A. M.; Ribeiro, A. H.; Pedregosa, F.; van Mulbregt, P.; Vijaykumar, A.; Bardelli, A. Pietro; Rothberg, A.; Hilboll, A.; Kloeckner, A.; Scopatz, A.; Lee, A.; Rokem, A.; Woods, C. N.; Fulton, C.; Masson, C.; Häggström, C.; Fitzgerald, C.; Nicholson, D. A.; Hagen, D. R.; Pasechnik, D. V.; Olivetti, E.; Martin, E.; Wieser, E.; Silva, F.; Lenders, F.; Wilhelm, F.; Young, G.; Price, G. A.; Ingold, G. L.; Allen, G. E.; Lee, G. R.; Audren, H.; Probst, I.; Dietrich, J. P.; Silterra, J.; Webber, J. T.; Slavič, J.; Nothman, J.; Buchner, J.; Kulick, J.; Schönberger, J. L.; de Miranda Cardoso, J. V.; Reimer, J.; Harrington, J.; Rodríguez, J. L. C.; Nunez-Iglesias, J.; Kuczynski, J.; Tritz, K.; Thoma, M.; Newville, M.; Kümmerer, M.; Bolingbroke, M.; Tartre, M.; Pak, M.; Smith, N. J.; Nowaczyk, N.; Shebanov, N.; Pavlyk, O.; Brodtkorb, P. A.; Lee, P.; McGibbon, R. T.; Feldbauer, R.; Lewis, S.; Tygier, S.; Sievert, S.; Vigna, S.; Peterson, S.; More, S.; Pudlik, T.; Oshima, T.; Pingel, T. J.; Robitaille, T. P.; Spura, T.; Jones, T. R.; Cera, T.; Leslie, T.; Zito, T.; Krauss, T.; Upadhyay, U.; Halchenko, Y. O.; Vázquez-Baeza, Y. SciPy 1.0: Fundamental Algorithms for Scientific Computing in Python. *Nat. Methods* **2020**, *17* (3), 261–272. https://doi.org/10.1038/s41592-019-0686-2.

(26) O'Boyle, N. M.; Morley, C.; Hutchison, G. R. Pybel: A Python Wrapper for the OpenBabel Cheminformatics Toolkit. *Chem. Cent. J.* **2008**, *2:5*. https://doi.org/10.1186/1752-153X-2-5.

(27) O'Boyle, N. M.; Banck, M.; James, C. A.; Morley, C.; Vandermeersch, T.; Hutchison, G. R. Open Babel: An Open Chemical Toolbox. *J. Cheminform.* **2011**, *3* (10), 239–245. https://doi.org/10.1186/1758-2946-3-33.

(28) Dos Santos, P. L.; Chen, D.; Rajamalli, P.; Matulaitis, T.; Cordes, D. B.; Slawin, A. M. Z.; Jacquemin, D.; Zysman-Colman, E.; Samuel, I. D. W. Use of Pyrimidine and Pyrazine Bridges as a Design Strategy to Improve the Performance of Thermally Activated Delayed Fluorescence Organic Light Emitting Diodes. *ACS Appl. Mater. Interfaces* **2019**, *11* (48), 45171–45179. https://doi.org/10.1021/acsami.9b16952.

(29) Galicia, M.; González, F. J. Electrochemical Oxidation of Tetrabutylammonium Salts of Aliphatic

Carboxylic Acids in Acetonitrile. *J. Electrochem. Soc.* **2002**, *149* (3), D46–D50. https://doi.org/10.1149/1.1450616.

(30) Hossain, A.; Engl, S.; Lutsker, E.; Reiser, O. Visible-Light-Mediated Regioselective Chlorosulfonylation of Alkenes and Alkynes: Introducing the Cu(II) Complex [Cu(Dap)Cl 2] to Photochemical ATRA Reactions. *ACS Catal.* **2019**, *9* (2), 1103–1109. https://doi.org/10.1021/acscatal.8b04188.

(31) Nakajima, M.; Fava, E.; Loescher, S.; Jiang, Z.; Rueping, M. Photoredox-Catalyzed Reductive Coupling of Aldehydes, Ketones, and Imines with Visible Light. *Angew. Chemie Int. Ed.* **2015**, *54* (30), 8828–8832. https://doi.org/10.1002/anie.201501556.

(32) Speckmeier, E.; Fischer, T. G.; Zeitler, K. A Toolbox Approach To Construct Broadly Applicable Metal-Free Catalysts for Photoredox Chemistry: Deliberate Tuning of Redox Potentials and Importance of Halogens in Donor − Acceptor Cyanoarenes. *J. Am. Chem. Soc.* **2018**, *140*, 15353–15365. https://doi.org/10.1021/jacs.8b08933.

(33) Belger, C.; Neisius, N. M.; Plietker, B. A Selective Ru-Catalyzed Semireduction of Alkynes to Z Olefins under Transfer-Hydrogenation Conditions. *Chem. - A Eur. J.* **2010**, *16* (40), 12214–12220. https://doi.org/10.1002/chem.201001143.

(34) Goti, A.; Cicchi, S.; Cacciarini, M.; Cardona, F.; Fedi, V.; Brandi, A. Straightforward Access to Enantiomerically Pure, Highly Functionalized Pyrrolizidines by Cycloaddition of Maleic Acid Esters to Pyrroline N-Oxides Derived from Tartaric, Malic and Aspartic Acids - Synthesis of (-)-Hastanecine, 7-Epi-Croalbinecine And (-)-. *European J. Org. Chem.* **2000**, No. 21, 3633–3645. https://doi.org/10.1002/1099-0690(200011)2000:21<3633::aid-ejoc3633>3.3.co;2-w.

(35) Zuo, Z.; Macmillan, D. W. C. Decarboxylative Arylation of α-Amino Acids via Photoredox Catalysis: A One-Step Conversion of Biomass to Drug Pharmacophore. *J. Am. Chem. Soc.* **2014**, *136* (14), 5257–5260. https://doi.org/10.1021/ja501621q.
